# Accurate Quantification of Single Aerosol Particle Microphysical Properties Using Broadband Light Scattering Spectroscopy

**DOI:** 10.1021/acs.jpca.6c00409

**Published:** 2026-04-16

**Authors:** Aidan Rafferty, Andrew J. Orr-Ewing, Jonathan P. Reid, Michael I. Cotterell

**Affiliations:** 1 Department of Chemistry, Physical and Theoretical Chemistry Laboratory, 6396 University of Oxford, South Parks Road, Oxford OX1 3QZ, United Kingdom; 2 School of Chemistry, 1980 University of Bristol, Cantock’s Close, Bristol BS8 1TS, United Kingdom

## Abstract

The wavelength-dependent refractive indices of aerosol particles, *n*(λ), are essential quantities for several areas of atmospheric science. Broadband light scattering (BLS) is a technique that has the potential to determine *n*(λ) and particle size at the single particle level by measuring variations in light scattering intensity with wavelength. However, a significant barrier to the use of BLS is the time-consuming analysis of measured spectra, caused primarily by the need to simulate large numbers of BLS spectra. We introduce a new approach to fitting BLS spectra for single, levitated, nonabsorbing aerosol particles that reduces the time required for analysis by minimizing the number of spectra that require calculation. The method is tested by comparing BLS measurements with concurrent cavity ring-down spectroscopy (CRDS) measurements of extinction cross section for two benchmark nonabsorbing aerosol species: (i) 1,2,6-hexanetriol (a semivolatile organic species) and (ii) aqueous particles containing the hygroscopic salt ammonium sulfate. The accuracy of the BLS-retrieved values for aerosol particle sizes and *n*(λ) is verified by their use in simulations that reproduce the simultaneously measured BLS spectra and CRDS-derived extinction cross sections. For particles of radius 0.8–2.5 μm interrogated in our experiments, particle radii and the real component of the complex refractive index are retrieved with typical precisions of 1.4 nm and ∼10^–3^, respectively, across the 380–800 nm wavelength range of the measurements.

## Graphical abstract

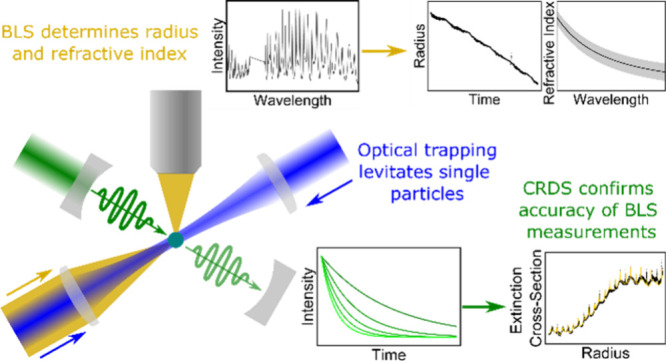

## 1 Introduction

Aerosols exert a profound impact on Earth’s climate and contribute to regulating its energy budget.^1–4^ Among the largest uncertainties in projecting future climate are those associated with the direct radiative forcing effect, i.e. how aerosol particles scatter and absorb radiation from short near-ultraviolet (near-UV) wavelengths to longer infrared (IR) wavelengths.^1,4,5^ Many factors contribute to these uncertainties, including poor characterization of the aerosol optical properties needed by climate models.^1^ A critical parameter for calculations of aerosol optical properties is the complex refractive index, *m* = *n* + *ik*, of an aerosol particle.^4^ Broadly, the real (*n*) and imaginary (*k*) parts determine how much light a particle scatters and absorbs, respectively.^4^ Both *n* and *k* depend on the wavelength of light with which an aerosol particle is illuminated. Often, atmospheric aerosol can be treated as nonabsorbing, meaning that *k* can be neglected. Therefore, models of aerosol climate effects can be improved by accurate characterizations of *n* from ultraviolet to infrared wavelengths.

Aerosol optical properties can be measured by studying either single particles or an ensemble of aerosol particles in a plume.^6,7^ Averaging effects present in ensemble methods mean that physical and chemical properties cannot be retrieved as precisely as with single particle methods. For example, Mason et al. found that refractive index measurements on single particles had uncertainties around an order of magnitude lower than ensemble experiments.^8^ Assessments of how uncertainties in refractive index propagate through to errors in radiative forcing efficiency (RFE) predictions reveal that even small uncertainties in *n* can lead to large uncertainties in RFE.^9,10^ An uncertainty in *n* as low as ± 0.003 can lead to a ± 5% uncertainty in RFE for typical particle sizes in the atmosphere, while an uncertainty of ± 0.02 leads to RFE uncertainties as high as 20%.^10^ The former uncertainty level in aerosol refractive index measurements has only been achieved using single particle methods. Therefore, the optimum method for quantifying aerosol optical properties should retrieve the refractive indices of single aerosol particles with high precision and accuracy across the broadest range of wavelengths possible.

There are several methods available for measuring optical properties at the single particle level. Angular light scattering (ALS), also known as phase function fitting, is used widely in the aerosol community.^11–15^ Particle radius and refractive index are determined using the pattern of dark and bright fringes in scattered laser light. While ALS can determine *n* with a precision of ∼10^–3^, it does so only at a single wavelength.^14^ An alternative approach is to use cavity ring-down spectroscopy (CRDS), which provides direct measurements of extinction cross sections.^16–19^ Single particle CRDS measurements enable highly precise retrievals of *n* with uncertainties ∼10^–4^.^14,20^ However, as with ALS, CRDS typically allows retrieval of the refractive index at a single wavelength only. Furthermore, CRDS cannot on its own disentangle the contributions of particle radius and refractive index to the measured cross sections, and therefore must be complemented by another technique to determine particle radii.^14,18,21^ Cavity-enhanced Raman spectroscopy (CERS) has also been used to retrieve single particle optical properties.^13,22–25^ CERS uses inelastically scattered light to excite Mie resonances, the positions of which are used to determine the particle radius and refractive index. The wavelength dependence of *n* can be determined over the relatively narrow wavelength range spanned by the Raman bands.^26^

In principle, broadband light scattering (BLS) is an ideal method for interrogating aerosol optical properties: it is applicable to single particles and makes measurements across a wide range of wavelengths. Despite being introduced almost 20 years ago, BLS has not been adopted widely by the aerosol community.^27^ One possible barrier to widespread adoption is the complex and time-consuming nature of analysis of BLS spectra. Early approaches used the spacing between resonances observed in BLS spectra to retrieve particle radii.^27–29^ However, the analysis methodologies of these early works relied on prior knowledge of the refractive index. More recent BLS studies instead use the measured wavelengths of the resonance peaks in a spectrum. Radius and refractive index are determined by minimizing the difference between measured and calculated resonance wavelengths through a grid search^30–32^ or via the MRFIT algorithm.^33–35^ However, there is potentially useful information in the widths and relative intensities of peaks that is not considered when only analyzing peak positions.^36^

An alternative approach fits the entire measured BLS spectrum. This approach typically uses a grid search, where ranges and resolutions are chosen for parameters needed to describe the particle radius and refractive index, and spectra are calculated for each combination of parameters. Calculated spectra are then compared with measured spectra to see which combination of parameters most accurately reproduces an observed spectrum. Grid searches typically start by using coarse resolutions in each parameter dimension, then increase their resolutions in subsequent steps to find precise parameters. Without prior knowledge of likely values for each parameter, grid searches require time-consuming evaluation of large numbers of spectra. The only reports of a grid search approach to full-spectrum fitting for nonabsorbing particles employed a lookup table of precalculated spectra with different parameter combinations in their analysis.^36–38^ While lookup tables avoid the need for repetitive calculations of large numbers of spectra, their success relies on having anticipated all possible parameter combinations prior to analysis, and storing so many spectra requires a large amount of computer memory. Grid searches have also been employed in studies on absorbing particles, but required prior knowledge of radius and/or refractive index measured by other means before optimization.^39–41^ Thus, there is currently no analytical framework for analyzing full BLS spectra in a manageable time without making potentially restrictive assumptions or performing prior calculations.

Motivated by the need for faster analysis of full BLS spectra, we present a retrieval framework designed to make full spectrum fitting computationally tractable without sacrificing precision. We demonstrate the approach for nonabsorbing single particles and assess accuracy using concurrent extinction measurements. Section 2 describes a new experimental platform for the simultaneous acquisition of BLS spectra and extinction cross sections for a single particle, using BLS and CRDS concurrently to interrogate an optically trapped aerosol particle. Section 3 reports a novel methodology for full spectrum analysis of BLS spectra for nonabsorbing particles to retrieve particle radius and wavelength-dependent refractive index values. Section 4 presents benchmark measurements of BLS spectra for: (i) the evaporation of single component particles comprised of 1,2,6-hexanetriol; (ii) the hygroscopic response of aqueous particles containing ammonium sulfate. The retrieved particle properties from BLS spectral analysis are validated by comparison with measured BLS spectra and CRDS-measured extinction cross sections. We summarize our findings and suggest research avenues employing these tools in Section 5.

## 2 Experimental Methods

Figure 1 summarizes our experimental setup. Optical trapping of single aerosol particles is achieved using a dual-beam optical trap. Light from a 473 nm wavelength laser (Gem 473, Laser Quantum) is expanded by a 11.4 × beam expander and split into two arms using a polarizing beam splitter. A half-wave plate before the beam expander adjusts the polarization of the beam to equalize the powers in both arms. The two laser beams are focused into a custom-built trapping cell from opposite sides using 30 mm focal length achromatic lenses. The two achromatic lenses are mounted such that they can be translated in three dimensions, allowing the counterpropagating beams to be focused to approximately the same point to facilitate trapping of single particles. The mounts can also be moved together in two dimensions perpendicular to the CRDS beam to translate a trapped particle into the center of the CRDS beam. Aerosol is produced by nebulizing bulk aqueous solutions of the desired solute (either 1,2,6-hexanetriol or ammonium sulfate) using a medical nebulizer (NE-U07, Omron) and introducing the particles produced into the air flowing through the trapping cell. The relative humidity (RH) inside the trapping cell is controlled by mixing two flows of nitrogen: one dry and the other passing over two water baths to humidify the flow. By controlling the relative flow rates of each, the RH in the cell is adjusted.

**Figure 1:**
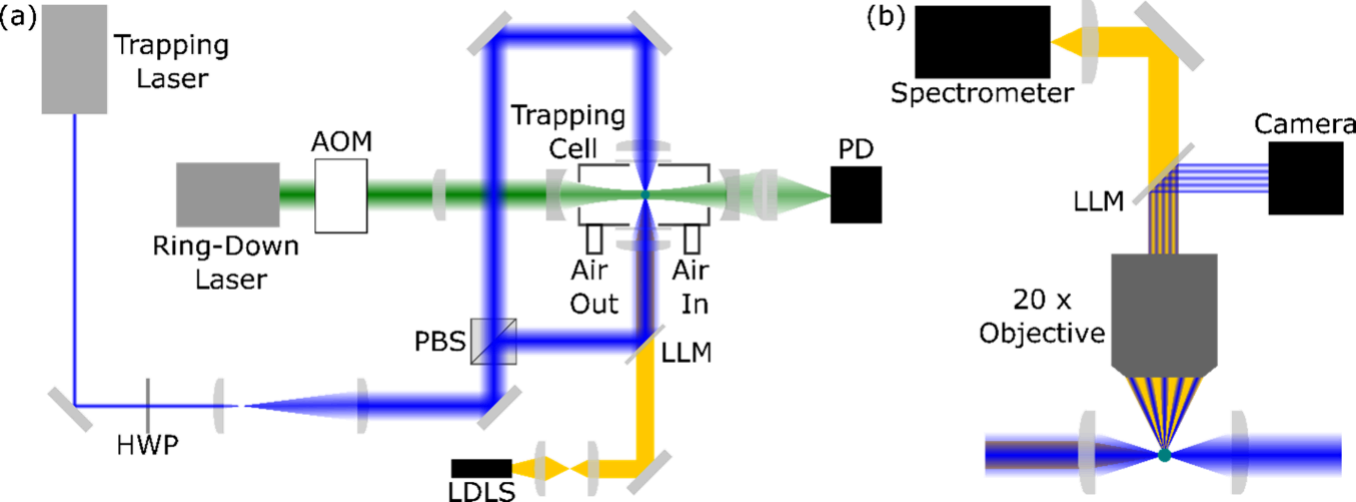
Schematic diagram of the BLS and CRDS setup used to perform measurements on trapped single particles. Panel (a) shows an overview of the optical setup. Panel (b) shows the BLS collection setup and is oriented perpendicular to the plane of panel (a). AOM: acousto-optic modulator; PD: photodiode; LDLS: laser-driven light source; PBS: polarizing beamsplitter; LLM: laser-line mirror; HWP: half-wave plate.

Broadband light from a laser-driven light source (EQ-99X-FC-S, Energetiq) is collimated using two achromatic lenses and directed onto one of the trapping lenses via a laser line mirror (NFD01-473-25x36, Semrock). Scattered light is collected perpendicular to the trapping beams by a 20× long working distance objective lens (MY20X-804, Mitutoyo). The elastically scattered laser light is directed by a laser line mirror (NFD01-473-25x36, Semrock) onto a CMOS camera (DCC1545M, Thorlabs) that records the phase function for visual inspection of particle trapping stability. The remaining elastically scattered light is passed onto a spectrometer (HR-2VN400, Ocean Insight) with a 25 μm slit width to measure BLS spectra. The integration time of the spectrometer is adjusted to attain an adequate signal-to-noise ratio and is typically in the range 20–50 ms. Consecutive spectra are then averaged to increase the signal-to-noise ratio of measured spectra and obtain a sampling rate of one spectrum per second. Simultaneously, we measure light extinction by the trapped particle using CRDS. The CRDS spectrometer used in this work has been reported in previous publications,^14,42–44^ and is described in detail in Section SI1 of the Supporting Information.

## 3 Analytical Framework

Our recent publication describes the algorithm used here for the efficient calculation of synthetic BLS spectra.^45^ Here, we describe how to utilize these spectral calculations to find the particle radius and wavelength-dependent refractive index from each measured BLS spectrum. We describe the refractive index using the effective oscillator model described by Bain et al.^22,33^ This model describes the refractive index of nonabsorbing species in the visible wavelength range using the tails of Lorentzian oscillators centered in the far-UV region:

$$n\left(\nu \right)=1+\frac{2}{\pi }\sum_{j=1}^{J}\frac{A_{j}\nu_{j}}{\nu_{j}^{2}-\nu^{2}}$$ 1

with ν = 1/λ the wavenumber, *A*_j_ the amplitude of the j^th^ oscillator, ν_j_ the central wavenumber of the j^th^ oscillator, and J the number of oscillators used to describe the refractive index. The effective oscillator model observes that the oscillator centered at the longest wavelength in the far-UV dominates the refractive index in the visible range. In this model, each chemical component of an aerosol particle requires one oscillator. For an aerosol particle comprising a single component, a single oscillator is required, and the amplitude and center wavenumber are simply denoted *A* and ν_0_, respectively.

Without prior knowledge of the particle radius or refractive index, a simple grid search over *r*, *A* and υ_0_ is too computationally intensive to be practical. For example, David et al. employed a grid search on measured BLS spectra to find radius and two Cauchy refractive index parameters using a lookup table of precalculated spectra. The dimensions of the approximate grid search alone required a library of over 800,000 spectra.^36^ Our recent publication on efficient generation of BLS spectra shows that spectra spanning the visible wavelength range for particles of radius 0.5–5 μm can be generated on time scales of around 0.25–2.5 s per spectrum on a desktop computer (Dell OptiPlex SFF Plus 7010 with 2500 MHz Intel Core i5–13,500 processor, the same computer platform as used in this work).^45^ Assuming that each spectrum can be calculated in one second, calculation of 800,000 spectra would take more than a week, and does not include the generation of subsequent spectra for the finer resolution steps of the procedure. Gathering measurement data sets, on the other hand, takes at most a few hours. This example illustrates an unavoidable aspect of full-spectrum analysis: the generation of synthetic spectra presents a bottleneck in BLS analysis of aerosol particle properties. While efficient generation of synthetic spectra is critically important, this alone does not allow particle parameter retrievals to be performed on reasonable time scales, meaning the number of spectra needed for full-spectrum analysis must also be minimized.

Our analysis process begins by isolating the wavelength-dependent Mie resonance structure in the measured spectra using the method described in Section SI2 of the Supporting Information. This process removes both the wavelength-dependent intensity of the light source and the slowly varying baseline component of the scattered spectrum, leaving only the Mie resonances. Thus, the corresponding baseline components of the calculated spectra must also be removed (see Section SI2 of the Supporting Information) prior to comparison with measured spectra. We break the analysis process into two steps. First, we perform an approximate search in which a BLS spectrum is divided into multiple wavelength windows, with each spectral window fitted separately to synthetic spectra calculated using wavelength-invariant refractive indices. The results from the first step provide approximate parameters used to generate synthetic spectra with wavelength-dependent refractive indices for the second fitting step. The first step reduces the required number of synthetic spectra to an amount that can be evaluated on a time scale of hours. Once synthetic spectra are calculated, the second step begins with calculating correlations between the measured and synthetic spectra with wavelength-dependent refractive indices obtained using parameters close to those determined in the first step. Precise values of radius and effective oscillator parameters are retrieved by interpolating between (*r*, *A*, υ_0_) combinations producing high correlation with observed spectra.

### 3.1 Finding Approximate Particle Parameters

This step of the procedure exploits the fact that *n* is approximately constant over a narrow wavelength range. We can use this fact to find values of *n* in narrow wavelength ranges across a measured spectrum and fit them to determine rough values for effective oscillator parameters, as well as a value of *r* that fits each window well. Search ranges for particle radius and refractive index, denoted *r*_i_ and *n*_i_, are first chosen to span likely values for the particle under consideration. Synthetic spectra are generated for each combination of *r*_i_ and *n*_i_, with *n*_i_ held constant across the full wavelength range of the spectra. The method for generating these spectra efficiently is described in Section SI3 of the Supporting Information. The rest of the approximate retrieval method is summarized in Figure 2. In line with previous work, we evaluate the similarity between measured and synthetic spectra using the Pearson sample correlation coefficient.^39^ For each value of *r*_i_ and *n*_i_, we evaluate the Pearson sample correlation coefficients between the measured spectrum and synthetic spectra in each spectral window, shown in Figure 2a for spectral windows of 500–560 nm, 560–620 nm, 620–680 nm, 680–740 nm and 740–800 nm (see below for discussion of these choices of intervals). We use two pieces of information from each correlation vs *n*_i_ plot to determine the approximate radius and effective oscillator parameters: *C*_λ_, the maximum correlation in a given spectral window; and *n*_λ_, the refractive index that produces *C*_λ_.

**Figure 2:**
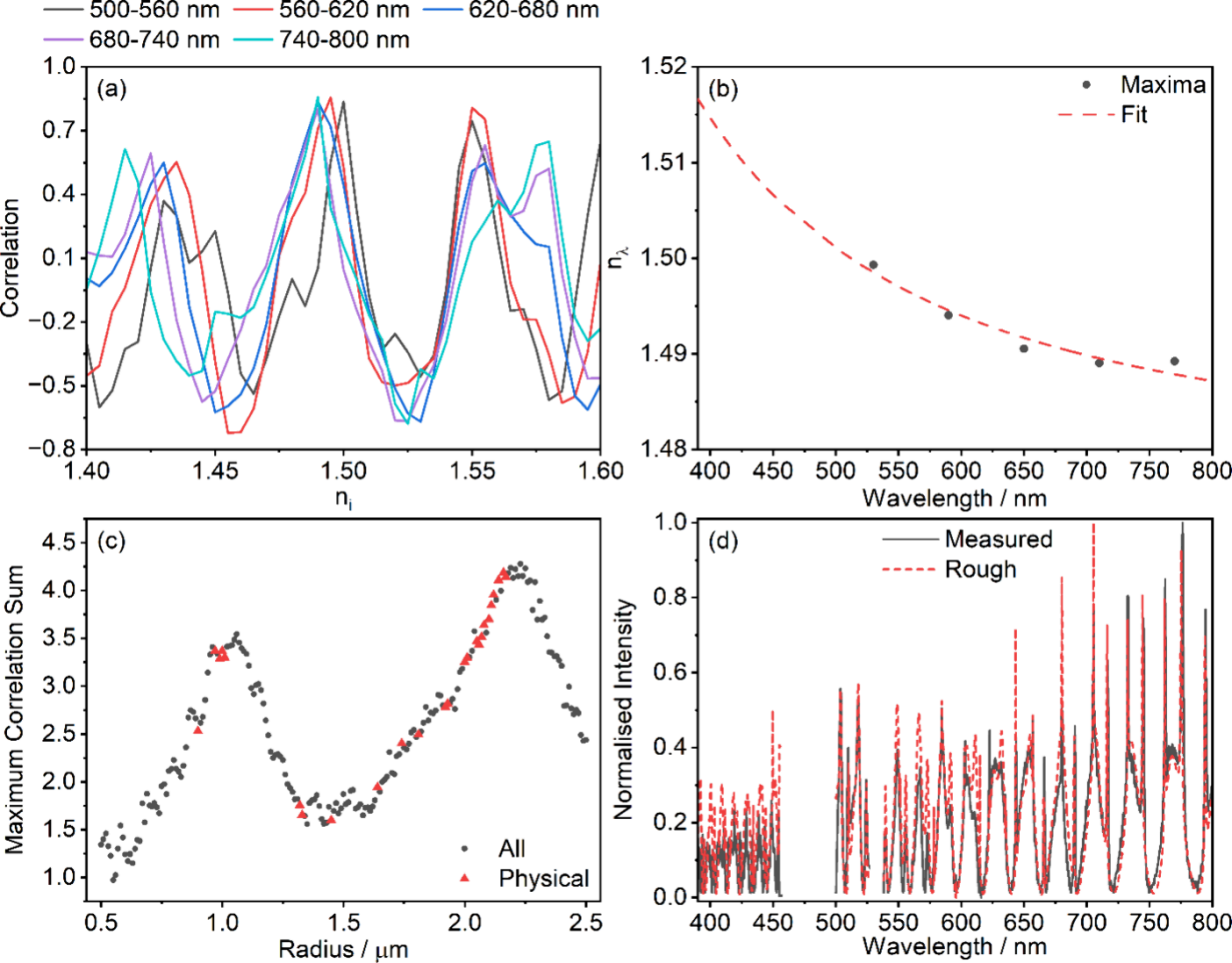
Overview of method for determining approximate particle parameters. (a) Variation in Pearson correlation coefficient for a single value of radius (*r*_i_ = 2.16 μm) as a function of real refractive index for the wavelength intervals indicated in the legend above the plot. (b) Highest correlation refractive index values refined by parabolic interpolation for each wavelength bin in panel (a) (black circles) and fit using eq 1 (red dashed line). (c) Sum of the maximum correlations in each wavelength interval as a function of radius (black circles), with red triangles showing radii producing a physically realistic set of refractive index points. (d) Comparison of experimental spectrum (solid black line) with spectrum generated using the approximate radius determined from the point of maximum correlation among the red triangles in panel (c) and the oscillator parameters from the fit in panel (b) (red dashed line).

To determine the approximate radius, we calculate the sum of *C*_λ_ for each *r*_i_ (black circles in Figure 2c). The logic underlying this step is that while several combinations of *r*_i_ and *n*_i_ can give a high correlation within a single wavelength interval, only values around the correct radius will produce high correlations in all wavelength bins. Thus, the highest sum of *C*_λ_ should correspond to a good approximation for the radius. Since we fit each wavelength interval independently of the others, there are no constraints on the relative values that the set of *n*_λ_ may take, potentially resulting in an unphysical set of *n*_λ_ values. Consistent with the normal dispersion exhibited by most nonabsorbing materials, we only consider *r*_i_ values for which the values of *n*_λ_ in the shortest and longest wavelength intervals are the largest and smallest of the set, respectively (red triangles in Figure 2c). The approximate radius, *r*_app_, is the value of *r*_i_ with the highest sum of *C*_λ_ and a physically realistic set of *n*_λ_, i.e. the red triangle with the greatest value in Figure 2c. The values of *n*_λ_ determined for *r*_app_ are refined using parabolic interpolation by applying a formula to (*n*_λ_, *C*) and the (*n*_i_, *C*_i_) points immediately before and after (*n*_λ_, *C*_λ_).^46^ Refined *n*_λ_ values are fitted to eq 1 to determine approximate oscillator parameters *A*_app_ and ν_app_ (Figure 2b). The wavelengths used when fitting *n*_λ_ are the midpoints of the corresponding wavelength intervals. A comparison between an experimental spectrum and one generated using the approximate particle parameters determined from panels (b) and (c) is shown in Figure 2d.

This methodology was tested extensively on synthetic data with added Gaussian noise representative of that in our experimental data. From these studies, we determined the best wavelength intervals to use were 500–560 nm, 560–620 nm, 620–680 nm, 680–740 nm and 740–800 nm. These intervals are chosen to be optimal for several competing factors. Broader wavelength intervals produce more prominent maxima as a function of refractive index (see Figure 2a) and allow the wavelength range of 500–800 nm to be spanned using fewer intervals, thereby reducing the time required for approximate parameter retrieval. However, the underlying assumption that the refractive index is constant across the interval is poorer for broad wavelength intervals, making retrieved parameters less accurate. Consideration of these factors led to the choice of 60 nm intervals. Intervals for wavelengths shorter than 500 nm were excluded because they tended to produce outlier values of *n*_λ_, leading to inaccurate retrievals of *A*_app_ and ν_app_. This poorer fitting is likely related to the underlying assumption that the refractive index is constant in each interval. Since the refractive index of a nonabsorbing material changes most rapidly at shorter wavelengths, this assumption is better satisfied at longer wavelengths. We found that the optimal resolution in *r*_i_ was 10 nm, and for *n*_i_ was 0.005. Finer resolution was not found to offer greater precision or accuracy in retrieved parameters, instead only increasing computational time. The relatively coarse resolution in both *r*_i_ and *n*_i_ makes this step of the procedure fast: generation of spectra in size parameter space for the necessary range of *n* values takes under a minute to complete, and approximate particle parameters are obtained in less than 0.5 s for each measured spectrum. Typically, this approach determines the radius to an accuracy of ± 20 nm for particles in the 1–2 μm range and the oscillator parameters are extracted with an accuracy of ± 0.5 μm^–1^ for typical values between 6 and 11 μm^–1^.

### 3.2 Finding Precise Particle Parameters

To find precise values for particle parameters, we first calculate a grid of spectra that include a wavelength-dependent refractive index (modeled using eq 1 for a single oscillator) to which experimental spectra will be compared. The limits of the search space are found from the approximate parameters determined via the method described in Section 3.1. Testing on synthetic data revealed that the minimum grid resolution required to achieve accurate results is 0.067 μm^–1^ for *A* and 0.02 μm^–1^ for ν_0_. Higher resolution in ν_0_ is required to ensure accurate results for peak positions determined by parabolic interpolation (as shown below), whereas fewer points in the *A* dimension are required to find the maximum correlation for a given radius. Spanning a range of ±0.5 μm^–1^ for each oscillator parameter with these resolutions produces 816 unique spectra that must be calculated for each value of particle radius in the search space.

The number of radii included in the grid search depends on how much the particle size changes during an experiment. In single-component evaporation measurements, such as for the semivolatile organic species 1,2,6-hexanetriol discussed in Section 4.1, the size change is governed by the vapor pressure of the particle and the duration of the experiment. The size change in hygroscopic systems, such as for the aqueous ammonium sulfate particles discussed in Section 4.2, is dictated by the hygroscopicity of the solute and the change in RH during the experiment. The largest radius change we observed in our experiments was ∼1 μm. Our studies on synthetic data revealed that we require 10 nm resolution in the radius dimension of the grid. Even in this extreme limit of particle size change, our reduced search space consists of ∼80,000 potential parameter combinations, around 10% of the size of the search space used for the first stage of the grid search in the work of David et al.^36^ Assuming, as above, that each spectrum takes 1 s to evaluate, the necessary calculations can be completed in ∼22 h. This estimate represents an upper extreme for our experiments and is far shorter than the >1 week time scale estimated above using the lookup table method of David et al. Typically the particle size will change by only a few hundred nanometers, making calculation of the necessary synthetic spectra much less intensive.

Furthermore, we take advantage of parallel computing to reduce the necessary computational time. Most modern desktop computers have multiple cores, allowing several calculations to run at the same time. We utilize seven cores on our computer, reducing computational time approximately 5-fold. For a spectrum generation time of one second, even the large search space described above is populated in around 4 h. As a specific example, the most intensive data set for 1,2,6-hexanetriol that we analyzed involved a particle with a radius that decreased by 1 μm from ∼2.35 μm to ∼1.35 μm. From the results of the approximate search, we required a search space of 102 radii, 16 values of *A* and 51 values of ν_0_, hence a total of 83,232 distinct spectra. These spectra were evaluated in 1 h and 55 min. Since the time for calculating a spectrum scales with particle size, search grids of similar dimension for smaller particles will require less computational time.

For each measured spectrum, the radius search space is found by smoothing the approximate radii found in Section 3.1 using a Savitzky-Golay filter (Figure S2) and taking the nearest radius grid point along with radii two grid points either side of this approximate radius. For each of these radii, we calculate the correlation between the measured and synthetic spectra for each (ν_0_, *A*) combination. Figure 3a shows a typical correlation map for a fixed value of radius as a function of *A* and ν_0_. This plot shows an obvious peak extending diagonally across (ν_0_, *A*)-space, from which we determine the maximum correlation *C*_max_ and the coordinates (ν_max_, *A*_max_) at which *C*_max_ occurs. To do so, the maxima occurring along the diagonal peak and their associated locations are found using parabolic interpolation^47^ at each value of *A* along the diagonal peak, producing the coordinates of the maxima (ν_m_, *A*_m_) indicated by the black circles in Figure 3a and their corresponding correlation values *C*_m_. The values of *C*_max_ and ν_max_ are found by fitting a smoothing spline (using the make_smoothing_spline function in the SciPy interpolate submodule) to *C*_m_ as a function of ν_m_ and finding the maximum (Figure 3b, red triangle). *A*_max_ is then found from linear interpolation between the (ν_m_, *A*_m_) points immediately before and after ν_max_ (Figure 3c, red triangle). Repeating this process for each radius in the reduced search space gives ν_max_, *A*_max_ and *C*_max_ as a function of radius, which are then interpolated to find precise particle parameters. To determine the precise parameters, we first perform parabolic interpolation on the *C*_max_ values to find the radius value at which the maximum correlation will occur (Figure 3d, blue diamond). This is our best-fit radius, *r*_fit_. ν_max_(*r*) and *A*_max_(*r*) are found by fitting a smoothing spline to each set of points. Evaluating these splines at *r*_fit_ gives ν_fit_ and *A*_fit_ (Figure 3e,f blue diamonds), respectively.

**Figure 3:**
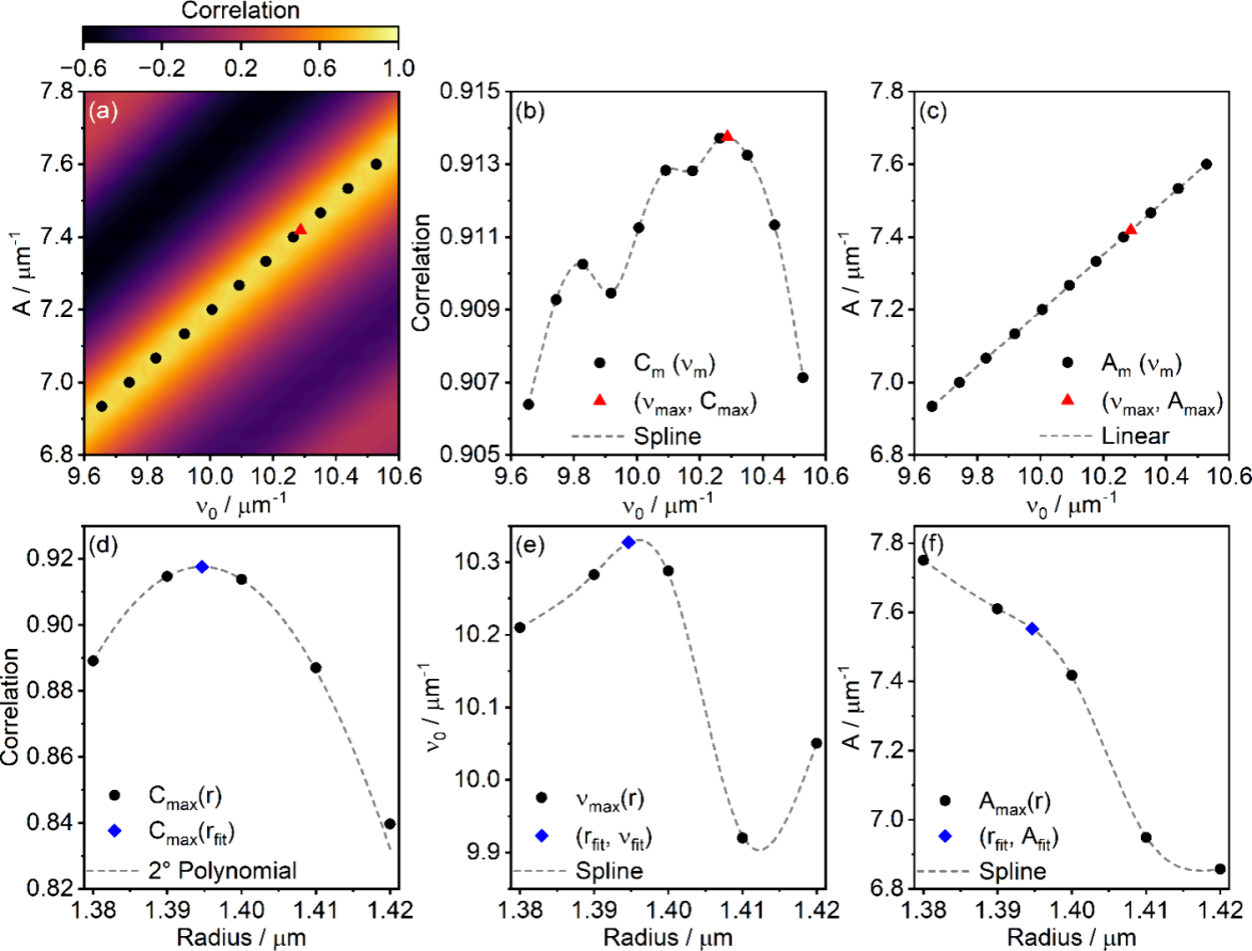
Summary of the precise parameter retrieval method. (a) A typical correlation map for one radius in the search space, with local maxima indicated by black circles and the global maximum determined from panels (b) and (c) shown by a red triangle. (b) Correlation values for the peaks in (a) (black circles), smoothing spline (gray dashed line), and maximum of fit (red triangle). (c) Linear interpolation (gray dashed line) of peak coordinates from panel (a) (black circles) with coordinates of the maximum correlation from panel (b) (red triangle). (d) Maximum correlations determined in panel (b) for each radius in the search space (black circles) with maximum as determined by parabolic interpolation (blue diamond). The second-degree polynomial (gray dashed line) is added to guide the eye. (e) ν_max_ as a function of radius (black circles) determined in panel (b), with smoothing spline (gray dashed line) and value at maximum radius from panel (d) (blue diamond). (f) *A*_max_ as a function of radius (black circles) determined in panel (c), with smoothing spline (gray dashed line) and value at maximum radius from panel (d) (blue diamond).

Although our interpolation scheme is complex, the required operations are computationally inexpensive, making this precise retrieval step quick. David et al. reported that their grid search method analyzed 25 spectra per hour (i.e., >2 min per spectrum).^36^ In contrast, our method typically requires 30–50 ms per measured spectrum (depending on the (ν_0_, *A*) search space). Therefore, once the necessary synthetic spectra are calculated, our method can analyze approximately 72,000 spectra in an hour. For the large data set mentioned above corresponding to a 1,2,6-hexanetriol particle, in which the radius changed by ∼ 1 μm and 12,035 spectra were acquired, fit parameters for all 12,035 measured spectra were retrieved in <10 min after generating the necessary synthetic spectra.

## 4 Results and Discussion

To evaluate the accuracy and precision of our BLS retrieval method, we apply the method to two commonly encountered situations in single particle aerosol experiments: (i) a single, semivolatile component evaporating over time, for which we use 1,2,6-hexanetriol, and (ii) a binary mixture of water and the nonvolatile inorganic solute ammonium sulfate changing size due to its hygroscopic response to a change in RH. Accuracy is investigated by comparing to measured BLS spectra, simultaneously measured extinction cross sections, and by comparison to previously reported measurements for these systems. The precision of retrievals is evaluated using the standard deviation in retrieved parameters across a single experiment.

### 4.1 Single Component Evaporation of 1,2,6-Hexanetriol

Figure 4 shows BLS results from an evaporating 1,2,6-hexanetriol particle. Panel (a) shows the processed experimental spectra as a function of time. The Mie resonance positions move to shorter wavelengths as the particle size reduces. The retrievals of the approximate fit parameters for this set of spectra using the parameter search method described in Section 3.1 are shown in panels (b)–(d), with the refined parameters obtained via the method of Section 3.2 shown in panels (e)–(g).

**Figure 4:**
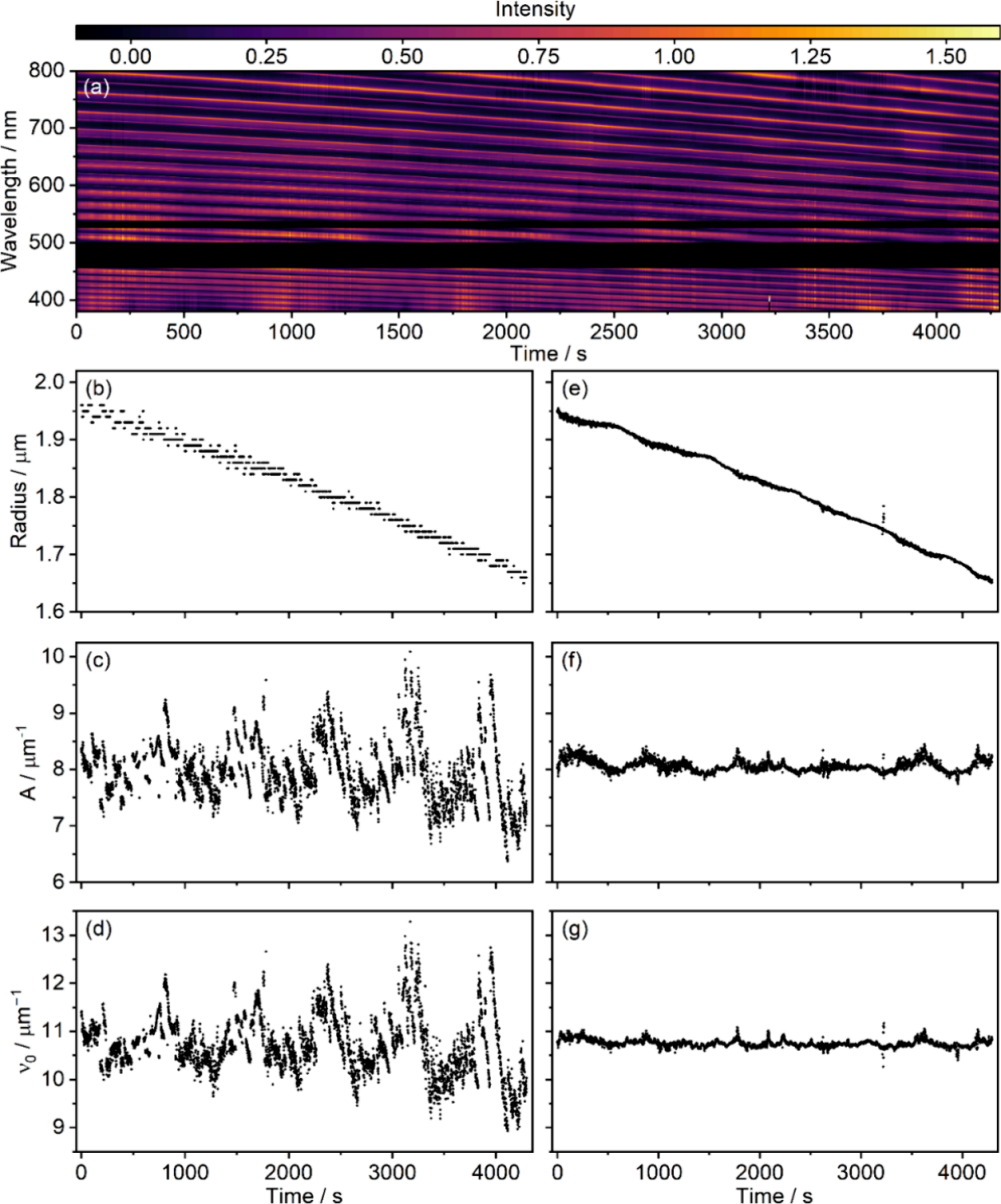
(a) Time-dependence of the Mie-resonance features in BLS spectra obtained from an evaporating 1,2,6-hexanetriol particle. Approximate (b) radius, (c) *A*, and (d) ν_0_ values determined using the procedure described in Section 3.1 and precise (e) radius, (f) *A*, and (g) ν_0_ values determined using the procedure described in Section 3.2.

We test the retrieval quality in two ways. First, we evaluate how well the measured spectra are reproduced by the best-fit parameters. Figure 5a shows a typical example, demonstrating agreement between the best-fit and measured spectra. This agreement is quantified using the sample Pearson correlation coefficient. Figure 5b compares correlations obtained (i) during the interpolation procedure, *C*_max_(*r*_fit_), described in Section 3.2, and (ii) by computing spectra using the best-fit parameters (*r*_fit_, *A*_fit_, ν_fit_). Correlations between measured and fitted spectra are high (mean values of 0.901 and 0.892 for (i) and (ii), respectively), indicating that the high level of agreement seen in Figure 5a is present across the entire data set. Additionally, the close agreement in correlations determined from the interpolation procedure and those calculated from best-fit parameters shows that those evaluated by interpolation are robust measures of fit quality.

**Figure 5:**
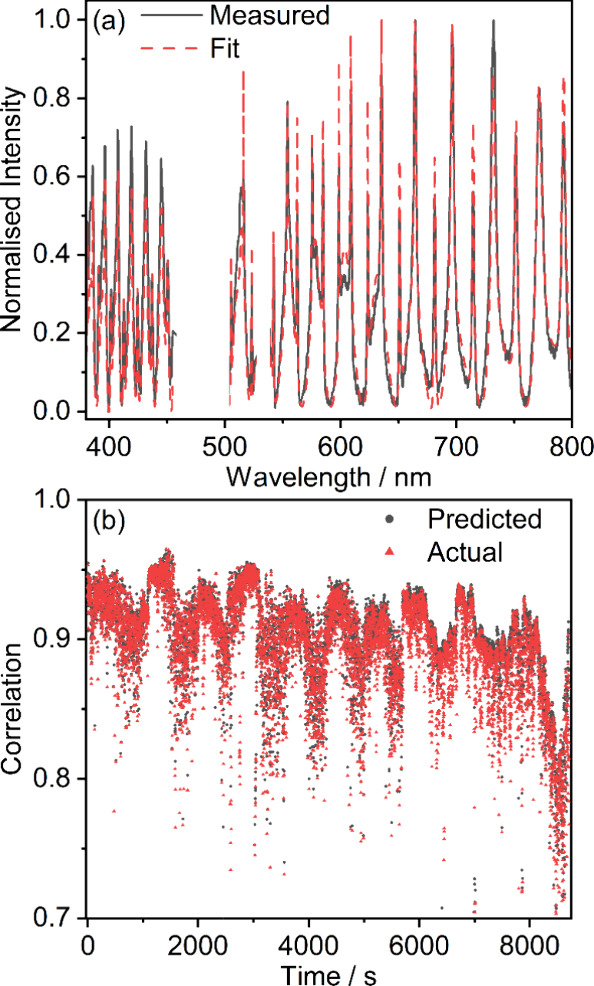
(a) Comparison between an experimental BLS spectrum (black line) and a simulated spectrum generated from fit parameters (red dashed line). (b) Correlation between measured and synthetic spectra predicted by the fitting procedure in Section 3.2 (black circles) and from a spectrum calculated using the retrieved parameters (red triangles).

We note that the retrieved radii in Figure 4e demonstrate weak oscillatory behavior as a function of time. Two mechanisms may explain this observation. As the droplet shrinks, its size periodically matches the resonance condition for supporting Mie resonances at the trapping laser wavelength that enhance heating and evaporation in a regular pattern.^13,48,49^ Moreover, radius-dependent oscillations in the trapped position can modulate the absorption cross section, producing periodic heating variations even without resonant effects.^50^

The second check of fit quality is to compare to simultaneously recorded CRDS extinction cross sections. Predicted values for extinction cross section can be calculated from Mie theory using the BLS-retrieved particle parameters of size and refractive index at the 532 nm wavelength of our CRDS spectrometer. Alternatively, the CRDS-measured ring-down times may be fitted directly to retrieve the refractive index using a method similar to previous work and described in detail in Section SI4 of the Supporting Information.^14,21,51,52^

Figure 6a shows an example of single-particle CRDS-measured extinction cross sections, the best-fit obtained using the grid search method described in Section SI4, and the extinction cross sections calculated using the BLS-retrieved particle radii and 532-nm refractive index. The similarity between the BLS- and CRDS-derived spectra is clear; the CRDS-retrieved refractive index of *n*(532 nm) = 1.4796 compares favorably with the value of 1.481 ± 0.005 obtained from analysis of BLS data for the same particle. The uncertainty in the latter value corresponds to one standard deviation propagated from the mean retrieved *A* and ν_0_ from all BLS spectra in the data set as detailed in Section SI5 of the Supporting Information. Figure 6b shows the retrieved *n* from replicate measurement CRDS and BLS data sets and demonstrates good agreement between the *n* values obtained by each technique, with almost all values agreeing within the uncertainties of the BLS retrievals. The mean precision of the individual measurements is 0.004, while the standard deviation between experiments is 0.005, suggesting that experiment-to-experiment variation in the BLS-retrieved values is as expected. The latter value is also similar to the standard deviation between the CRDS measurements, which is 0.004.

**Figure 6:**
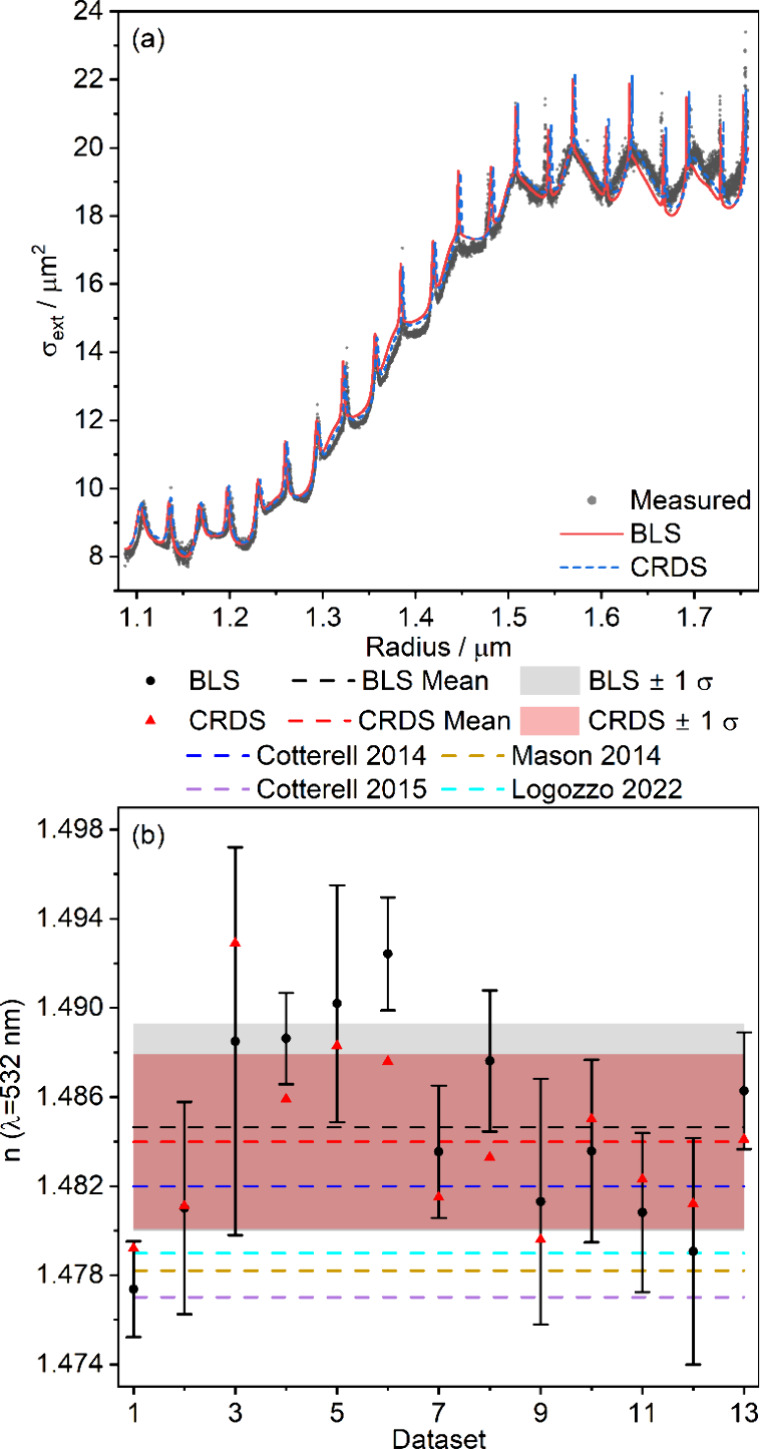
(a) Measured extinction cross sections (gray circles) for an evaporating 1,2,6-hexanetriol droplet, with fit (dashed blue line) and predictions from BLS measurements of *n* (solid red line). (b) Comparison of 532-nm refractive index values from replicate BLS measurements (black circles) and CRDS measurements (red triangles) with their means and standard deviations (dashed lines and shaded areas of the same color, respectively). Also shown are previous measurements from refs 19 (dark blue dashed line),^21^ (purple dashed line),^53^ (light blue dashed line), and^54^ (orange dashed line).

Our retrievals compare well with previously reported values for 1,2,6-hexanetriol, also shown in Figure 6b. The 532-nm refractive index of 1,2,6-hexanetriol has been measured in other single particle experiments, with values ranging from 1.477–1.482.^19,21,53,54^ Our retrieved values are slightly higher than previous estimates; but with a maximum difference of only 0.008 between our mean BLS-derived value of 1.485 and the minimum of the previously measured values 1.477, this difference could result from a small concentration of impurities. Further comparison can be made between our retrieved oscillator parameters and previous measurements. Logozzo et al. measured oscillator parameters for 1,2,6-hexanetriol of *A* = 7.78 μm^–1^ (at 290 K, the temperature of our laboratory) and ν_0_ = 10.67 μm^–1^ using CERS.^53^ Figure S3 shows that our mean BLS-retrieved values of *A* = 7.76 ± 0.20 μm^–1^ and υ_0_ = 10.53 ± 0.18 μm^–1^ both agree within error, reinforcing the quantitative agreement between parameters determined here and in previous studies.

### 4.2 Hygroscopic Response of Ammonium Sulfate

Experiments on single aerosol particles frequently look at systems containing more than one component. The most common example is the measurement of uptake and loss of water from a particle containing a hygroscopic species. The presence of two components and their evolving relative concentrations makes the description and retrieval of refractive index more complicated than for single component evaporation considered in Section 4.1. Here, we investigate the use of our fitting approach for hygroscopic response experiments on binary particles containing ammonium sulfate.

#### 4.2.1 Refractive Index of Binary Particles

We begin by describing a simplified model for the refractive index of a binary mixture. The effective oscillator model (eq 1) for the refractive index of a two-component system consisting of a solute (s subscripts, in our case ammonium sulfate) and water (w subscripts) gives

$$n=1+\frac{2}{\pi }\left(\frac{\varphi_{s}A_{s}\nu_{s}}{\nu_{s}^{2}-\nu^{2}}+\frac{\varphi_{w}A_{w}\nu_{w}}{\nu_{w}^{2}-\nu^{2}}\right)$$ 2

with ϕ_i_ the relative density of component i in the particle defined by φ_i_ = ρ_i_/ρ_pure_, in which ρ_i_ is the mass concentration of component i and ρ_pure_ is the density of a solution containing only this component. We calculate all densities using the parametrization of Tang et al.^55^ ρ_pure_ for water and ammonium sulfate are obtained by setting the mass concentration of ammonium sulfate to 0 and 1 in this parametrization, respectively.

The effective oscillator model assumes that the oscillators are centered far from the range of wavelengths that measurements span. If the center wavenumbers of each oscillator are similar and can be treated as a single, effective wavenumber, i.e. ν_*s*_ ≈ ν_*w*_ = ν_*e*_, eq 2 simplifies to

$$n=1+\frac{2}{\pi }\frac{\left(\varphi_{s}A_{s}+\varphi_{w}A_{w}\right)\nu_{e}}{\nu_{e}^{2}-\nu^{2}}=1+\frac{2}{\pi }\frac{A_{e}\left(\varphi_{s}\right)\nu_{e}}{\nu_{e}^{2}-\nu^{2}},$$ 3

which is a single effective oscillator with a composition-dependent amplitude, *A*_e_(φ_s_). Treating the refractive index of aqueous ammonium sulfate using eq 3 allows us to use the same retrieval process as for 1,2,6-hexanetriol, but with *A*_e_ expected to vary with particle size. Therefore, the search range for *A*_e_ needs to be extended: we found taking the mean of the approximate retrievals (using the process described in Section 3.1) for *A*_e_ ± 1.0 μm^–1^ was sufficient to fit BLS spectra for aqueous ammonium sulfate particles. However, the range may need to be extended for solutes that are particularly hygroscopic or have especially high refractive indices. Additionally, we found it best to take the mean of the approximate values of ν_e_ ± 0.6 μm^–1^. These considerations produce 1891 oscillator parameter combinations per radius in the search space. To illustrate an upper limit on computational processing time, we consider our data set corresponding to the largest aqueous ammonium sulfate particle as an example for which the particle spanned radii from 1.5–2.2 μm, requiring 70 radii in the search space after finding approximate parameters. We required ∼3 h to generate all the required spectra for the final stage of fitting. Since each radius now has more combinations of oscillator parameters to consider, calculating the correlations needed for interpolation also takes longer. Typically, a single measured spectrum was fitted in 80–100 ms. Nonetheless, the precise retrieval stage of the fitting procedure was completed in only 11 min for the 7635 measured spectra of the experiment.

Figure 7 compares retrievals from aqueous ammonium sulfate BLS spectra using the full spectral range of 380–800 nm and a reduced spectral range of 380–600 nm. Here, the changes in particle radius and refractive index are caused by the controlled change in the RH of the environment in which the particle is levitated, with controlled cycles between high (nominally 80%) and low (nominally 40%) RH values as shown in Figure 7a. We discuss the retrievals from the different spectral ranges further below, but both retrievals are consistent with the arguments used to derive eq 3: *A*_e_ varies significantly with water uptake/loss, while ν_e_ remains fairly constant (although small changes correlated with particle size are present). This outcome indicates that, despite its simplicity, eq 3 is suitable for describing the refractive index of this binary mixture. Furthermore, the precisions in the retrieved parameters are comparable to, or slightly better than, those for 1,2,6-hexanetriol. In particular, the mean precisions in *r*, *A* and υ_e_ for individual ammonium sulfate measurements were 1.9 nm, 0.16 μm^–1^, and 0.21 μm^–1^, respectively. The precisions in *A*_e_ and υ_e_ produce an uncertainty in refractive index at 532 nm of 9 × 10^–4^ using the method described in Section SI5 of the Supporting Information.

**Figure 7:**
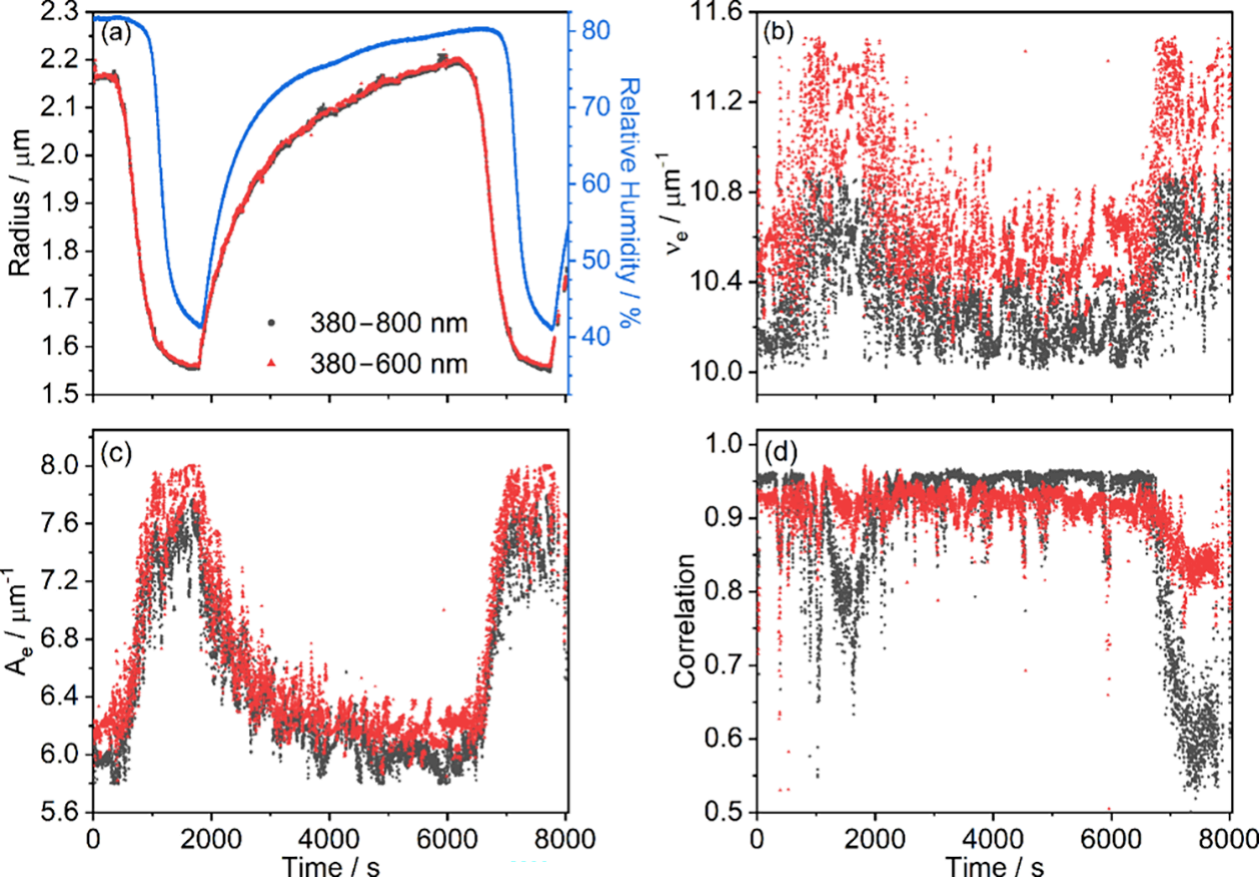
(a) Radius, (b) ν_e_, and (c) *A*_e_ retrieved from a typical BLS experiment on a levitated aqueous ammonium sulfate particle using the fitting method described in Section 3, along with (d) predicted correlations. Black circles indicate fits using the 380–800 nm wavelength range, and red triangles indicate fits using 380–600 nm. The relative humidity in the trapping cell exhaust is shown by the blue points in panel (a).

When fitting the full spectral range, the Pearson correlation coefficients show a trend with particle radius (Figure 7d): the predicted correlations are generally around 0.95 at large particle radii but fall to <0.8 for the smallest particle radii. This trend was observed to some degree across all our experiments, with Figures S5 and S6 providing insight. Figure S5 shows example spectra and their corresponding fits with high and low correlations. The peak positions of the measured and fitted spectra agree well in both cases, indicating that accurate parameters are retrieved. The lower value of correlation for Figure S5b arises because the intensities of the peaks at longer wavelengths are significantly lower in the measured than the fitted spectrum. These two observations, namely similar peak positions and reduced intensity, are characteristic of weak absorption by the particle.^39^ Moreover, Figure S6 compares simulated spectra with various values of *k* with the measured spectrum in Figure S5b. One can infer that *k* varies across the 600–800 nm range with values on the order of 10^–3^, although rigorous determination is beyond the scope of our work here. The unexpected absorption is likely due to an impurity, with iron – known to form a variety of colored complexes – listed as a potential impurity in our ammonium sulfate. Alternatively, this band could be a high-frequency overtone of a vibrational mode (the third overtones of the N–H and O–H stretching bands, if excited, would appear in this wavelength region). The presence of this band is the likely reason for the correlation trends seen in Figure 7d and the spectra in Figure S5.

The above example illustrates a distinct advantage of using full-spectrum fitting rather than fitting only peak positions. The peak positions match well regardless of whether absorption is occurring and analyzing these positions only would not have revealed a contribution from absorption, while absorption is clear from the full spectra including peak intensities. We can mitigate absorption effects by excluding parts of the spectrum where absorption occurs, although we note – and discuss further below – that an additional absorption feature may affect *n* even when *k* becomes negligible. Figure 7 also shows retrieved values from spectra spanning the reduced wavelength range 380–600 nm. Figure 7b,c shows that the values of the oscillator parameters increase by a small amount. However, the biggest difference is that the correlation remains relatively constant throughout the experiment (Figure 7d), rather than experiencing large dips at high solute concentrations, with the small decrease persisting at the end of the experiment less prominent than when fitting the full wavelength range. For the remainder of this work, results shown for ammonium sulfate are determined by fitting spectra over the reduced wavelength range 380–600 nm.

#### 4.2.2 Effective Oscillator Parameters for Ammonium Sulfate

To compare our retrievals to previously published results we must extract effective oscillator parameters for the pure solute, i.e. a hypothetical particle consisting only of ammonium sulfate in a theoretical liquid melt state. Extracting these effective oscillator parameters enables comparison between our results and others, with the potential to also be used in mixing rules for predicting the refractive index of mixed particles. In our experimental apparatus, we have a RH sensor placed in the exhaust from the trapping cell ∼10 cm downstream from a trapped particle. RH measurements at this position are not accurate representations of the RH at the particle position, precluding a straightforward deduction of the relative contributions of water and solute to refractive index using well-established hygroscopic growth parametrizations for aqueous ammonium sulfate particles. This is easily seen in Figure 7a, which shows a clear delay between the particle changing size in response to RH changes and the probe registering these changes. Therefore, an alternative approach is required to determine the effective oscillator parameters for the pure liquid melt solute. Section SI6 of the Supporting Information describes this alternative approach, and shows we require three fitting parameters: *w*_i_, the weight fraction of ammonium sulfate in the particle at the start of the experiment; *A*_s_, the amplitude of the effective oscillator for the pure solute; and ν_s_, the central wavenumber of the effective oscillator for the pure solute.

Values of *A*_s_, ν_s_ and *n* determined using this method are shown in Figure 8 and compared to values reported for single particles by Bain et al. using CERS.^22^ Uncertainties shown for each data set are the standard deviations in the corresponding *A*_e_ and ν_e_ from which *A*_s_ and υ_s_ are determined. As with 1,2,6-hexanetriol, our determinations of oscillator parameters agree with previously reported values within uncertainties. Our predictions for the wavelength-dependent refractive index of pure ammonium sulfate also agree with Bain et al. within error. The uncertainty in refractive index for the pure solute melt should be comparable to the precision of the individual measurements, namely 0.0009 as determined above. However, the uncertainty in the pure solute melt refractive index based on the standard deviation across all experiments is 0.02. The higher level of uncertainty is likely due to a correlation that exists between retrieved values of *w*_i_ and *A*_s_: in the fits, larger values of *A*_s_ can be compensated by a lower value of *w*_i_, giving similar refractive index values for a range of parameter combinations. We explore this correlation further below.

**Figure 8:**
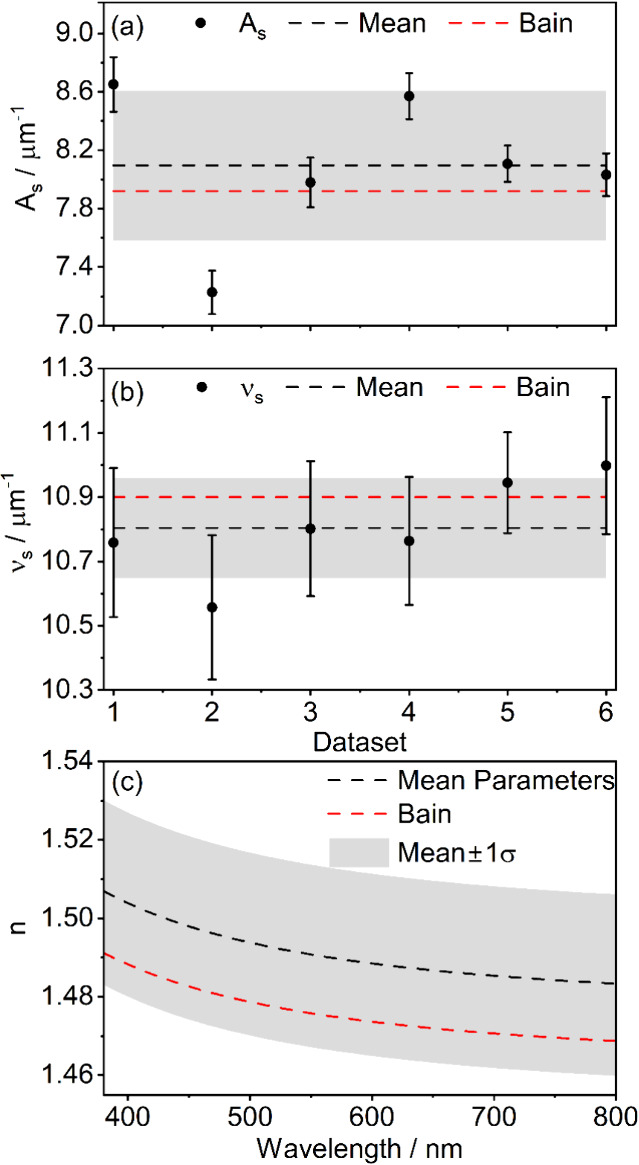
Retrieved values from replicate BLS measurements of (a) *A*_s_, and (b) ν_s_ for ammonium sulfate (black circles), along with their means (black dashed lines) and standard deviations (gray shaded areas). Values are compared to those from ref 22 (red dashed lines). (c) Refractive index (black dashed line) and uncertainty (gray shaded area) calculated from the mean oscillator parameters in panels (a) and (b), with comparison to the predicted values of Bain et al. (red dashed line).^22^

Similar to the comparisons shown in Section 4.1, we can validate the accuracy of our BLS fit parameters by comparing to the simultaneously recorded CRDS measurements. Since CRDS measures the refractive index at a single wavelength, two oscillator parameters cannot be extracted from the data as the contribution from dispersion cannot be disentangled from the contribution of *A*_s_. However, the refractive index at the CRDS wavelength for “pure” ammonium sulfate will be a constant. Thus, the CRDS data may be fitted using a simplified version of eq 2:

$$n_{532}=1+\varphi_{s}\left(n_{532}^{s}-1\right)+\varphi_{w}\left(n_{532}^{w}-1\right),$$ 4

in which φ_s_ and φ_w_ are predicted by assuming an initial weight fraction of ammonium sulfate as described in Section SI6, $n_{532}^{s}$ is the hypothetical refractive index of pure amorphous ammonium sulfate at the CRDS wavelength of 532 nm, and $n_{532}^{w}$ is the refractive index of water at 532 nm from ref 56. CRDS spectra are fitted using a grid search over *w*_i_, $n_{532}^{s}$ , and the intracavity beam waist, *w*_0_, similar to that described in Section SI4 but with the additional parameter *w*_i_.

Figure 9 compares the 532-nm refractive indices obtained from the BLS retrievals and by fitting CRDS measurements using a grid search. In the experiment shown in Figure 9a, the particle was subject to one dehumidifying cycle (RH changed from ∼80 to 40%), a humidifying cycle (∼40 to 80%), then a further dehumidifying cycle to below the efflorescence RH, meaning the radius range of the experiment was swept three times (with the exception of data below *r* ≈ 1.28 μm, which was only reached when the particle was fully dried). Measured cross sections from each cycle are overlaid in Figure 9a. Comparing each cycle emphasizes the quality of the measurements and the reproducibility of the radius fits; were the radius fits different from one another, the peaks in the CRDS measurements would shift between cycles. Figure 9a demonstrates that any shifts between cycles are within the noise of the measurement. The reproducibility of the refractive index fits is seen through examining the red line in Figure 9a. As with the experimental points, the fits from each cycle are plotted on top of one another. While a little variation between cycles can be seen, and is most obvious at larger radii, the reproducibility between cycles is good.

**Figure 9:**
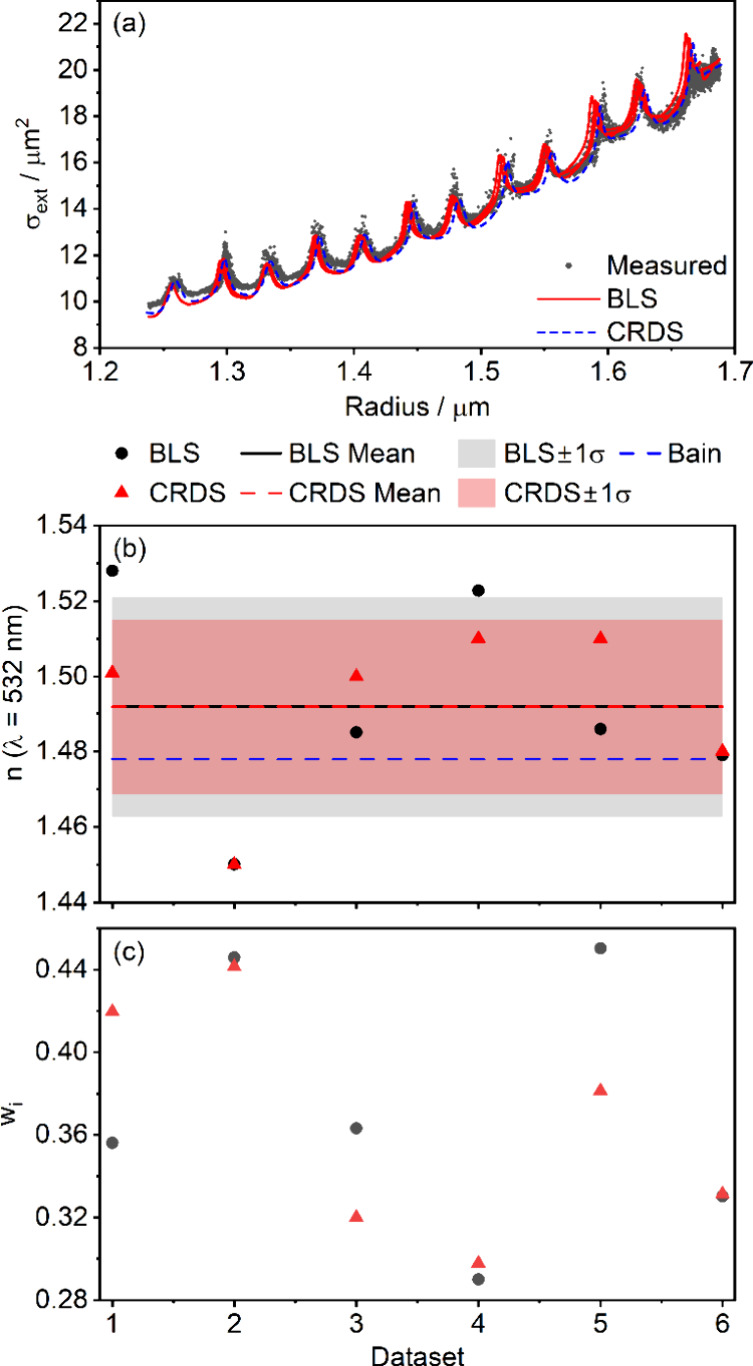
Comparison of the analysis of BLS and CRDS data for an aqueous ammonium sulfate droplet. (a) Measured (black circles) and simulated extinction cross sections using BLS-derived effective oscillator parameters (solid red line) and grid search fitting of CRDS data (dashed blue line). (b) Comparison between 532-nm refractive index values for pure ammonium sulfate determined from BLS-derived effective oscillator parameters (black circles) and grid search fitting of CRDS data (red triangles), along with their means (solid black and dashed red lines, respectively) and standard deviations (gray and red shaded areas). Also shown are values from a previous measurement (dashed blue line).^22^ (c) Initial weight fractions corresponding to the fitted values in panel (b) using BLS (black circles) and CRDS (red triangles).

While results within a given experiment are highly reproducible, Figure 9b shows a large amount of variation in $n_{532}^{s}$ , both between replicate experiments and between the BLS and CRDS measurements for a single droplet. As noted above, the values of *w*_i_ and *A*_s_ are correlated. This interdependence carries through into the 532 nm values of *n*, as seen by comparing Figure 9b and Figure 9c. Whichever of the measurements (BLS or CRDS) produces the lower value of *w*_i_ also produces the higher value of *n* in Figure 9b. Further evidence of this interdependence is shown by Figure 9a,b; the former compares the BLS and CRDS fits for data set 3, demonstrating almost perfect agreement between the two. However, Figure 9b shows that the values of $n_{532}^{s}$ retrieved by BLS and CRDS fitting of data set 3 differ by 0.015. This difference should result in large discrepancies between spectra for a constant *w*_i_, as seen in Figure S7, rather than the agreement seen in Figure 9a. The agreement between the BLS- and CRDS-derived extinction cross sections is attributed to the compensating effects of the different values of *w*_i_ retrieved by each method (0.363 and 0.320 for BLS and CRDS, respectively). The large spreads in retrieved values of *A*_s_ and $n_{532}^{s}$ likely have the same cause, although this cannot be proven directly. Given that the high standard deviation between experiments is a result of being unable to fully disentangle the contributions of *w*_i_ and either *A*_s_ (Figure 8) or $n_{532}^{s}$ (Figure 9), the precision in retrieved *n* for measurements on individual particles of 0.0009 is likely the better indicator of the performance of our fitting algorithm.

While difficulties in separating optimal values of *w*_i_ and *A*_s_ can explain the large uncertainties in *n*_532_^s^, the interdependence of these parameters does not explain why the means of both the BLS-derived and CRDS-derived values measured here are higher than previous measurements by ∼0.015. This discrepancy is likely due to the absorption band present at longer wavelengths identified above. The real and imaginary parts of the refractive index are linked through the Kramers–Kronig relations, and the presence of an additional absorption band (i.e., an additional oscillator in the framework of the oscillator model) will lead to modification of the real part of the refractive index, even at wavelengths where absorption is negligible. Thus, while absorption seems to be negligible at wavelengths below 600 nm, its effect on the real part of the refractive index may not be, potentially accounting for the discrepancy between our measured values and previous measurements.

## 5 Conclusions

This work advances single aerosol particle characterization by combining broadband light scattering (BLS) and cavity ring-down spectroscopy (CRDS) to interrogate a single optically trapped particle. This measurement approach provides temporally evolving BLS spectra over the wavelength range 380–800 nm alongside independent extinction cross section measurements, enabling validation of particle radius and refractive index retrievals. We report a new analysis framework for fitting BLS spectra for nonabsorbing aerosol particles that is computationally fast and delivers accurate and precise retrievals of particle radius and refractive index. Our two-stage analysis first obtains estimates of radius and effective oscillator parameters from constant refractive index fits to narrow spectral windows, before using these estimates to generate a grid of spectra from which to interpolate to precise values. By minimizing the number of synthetic BLS spectra that must be calculated, multithousand-spectra measurement data sets can be analyzed in only a few hours on a modern workstation.

We validate the methodology on two benchmark aerosol species that are commonplace in single-particle studies: evaporation of 1,2,6-hexanetriol and the hygroscopic growth/evaporation of aqueous ammonium sulfate. Across the 1,2,6-hexanetriol data sets, the measured and best-fit BLS spectra show consistently high correlations, and the BLS-predicted extinction cross sections agree with CRDS-derived values within experimental uncertainty, demonstrating that the retrieved radii and refractive indices are accurate. The method achieves nanometer-level particle sizing (with fractional standard deviations in particle radii of ∼0.1%) and typical precision in the refractive index of 0.004 across the measured 380–800 nm wavelength range of our spectra, rivaling or exceeding the best figures reported for other single particle optical techniques. Our analysis also tests the application of a single effective oscillator model to treat the refractive indices of binary mixtures of aqueous ammonium sulfate. This model captures the RH-dependent refractive index of aqueous ammonium sulfate with a composition-dependent single oscillator, thereby avoiding a costly expansion of the search space that would necessitate multiple oscillators, and produces similar precisions in retrieved parameters to 1,2,6-hexanetriol (average uncertainties in radius and refractive index of 1.9 nm and 9 × 10^–4^, respectively). We identify a weak, long-wavelength absorption feature (most likely stemming from impurities) that reduces best-fit correlations when water activity is low. Restricting our fitting to the nonabsorbing short-wavelength portion of the spectra improves retrievals. This case study emphasizes the value of full-spectrum fitting: intensity information can identify absorption bands that peak-position fitting approaches would miss.

Compared to previous work fitting full spectra of nonabsorbing particles, our approach is significantly faster and does not require the use of large lookup tables, giving our approach a marked advantage. A full comparison with peak position-based fitting, particularly with MRFIT, is beyond the scope of the work presented here. However, the results produced by our approach are comparable to MRFIT in terms of accuracy and precision, producing radii with precisions of 1–2 nm and refractive indices with precision on the order of 10^–3^. Typical processing times for data sets comprising thousands of spectra are a few hours and are comparable to a typical MRFIT workflow. Our approach benefits from eliminating the need to refine measured peak positions, which can take a similar time to the final fitting step using MRFIT. The computational time required to generate a synthetic BLS spectrum scales with particle size; thus, with all other variables held constant, our full spectrum approach becomes slower when considering larger particles. No such scaling exists for MRFIT, making it certain to be faster at larger particle sizes than those considered here. However, smaller particles tend to have broader modes, leading to greater uncertainty in the measured peak positions and the resulting parameters. This indicates that for smaller particles our approach would be preferable, with the best choice guided by pragmatism and experience. Further work could explore this comparison more thoroughly. Extension of the full-spectrum approach outlined here to light-absorbing particles, such as brown carbon-containing aerosol that presents high uncertainties in current climate models, should also be explored.^1,57,58^ The most efficient way to analyze spectra of absorbing particles is currently an open problem, and one to which full-spectrum analysis should be amenable.

## Supplementary Material



## Data Availability

The Python code written to analyze the data presented here is available on GitHub.^59^ Enquiries related to the data presented here should be addressed to Aidan Rafferty (aidan.rafferty@mail.mcgill.ca) or Michael Cotterell (michael.cotterell@chem.ox.ac.uk).

## Notes

Description of cavity ring-down spectrometer; description of background subtraction method; generation of spectra with wavelength-invariant refractive index; fitting of cavity ring-down spectroscopy data; precision of retrieved radius and effective oscillator parameters; retrieval method for effective oscillator parameters of ammonium sulfate; background subtraction example; example rough particle radius versus time with smoothing; retrieved oscillator parameters for 1,2,6-hexanetriol; uncertainties in parameter retrievals for 1,2,6-hexanetriol; example BLS spectra for aqueous ammonium sulfate droplets; BLS spectra including weak absorption; extinction cross sections with varying refractive indices (PDF)

## ‡

Department of Atmospheric and Oceanic Sciences, McGill University, 805 Sherbrooke Street West, Montreal, Quebec H3A 0B9, Canada

## Abbreviations

BLS: broadband light scattering
RFE: radiative forcing efficiency
ALS: angular light scattering
CRDS: cavity ring-down spectroscopy
CERS: cavity-enhanced Raman spectroscopy
AOM: acousto-optic modulator
UV: ultraviolet
IR: infrared.
RH: relative humidity
