# Supplementary material for: Accurate Quantification of Single Aerosol Particle Microphysical Properties Using Broadband Light Scattering Spectroscopy
Source: J Phys Chem A. 2026 Apr 16;130(17):3511–24. doi: 10.1021/acs.jpca.6c00409 (PMC13137246; doi:10.1021/acs.jpca.6c00409)
Supplement: Supplementary file 1 [file jp6c00409_si_001.pdf]

Supporting Information for "Accurate Quantification of Single Aerosol Particle Microphysical Properties using Broadband Light Scattering Spectroscopy"

Aidan Rafferty,[1,,‡] Andrew J. Orr-Ewing,[2] Jonathan P. Reid [2] and Michael I. Cotterell[1,*]*

[1] *Department of Chemistry, University of Oxford, Physical and Theoretical Chemistry Laboratory, South Parks Road, Oxford, OX1 3QZ, United Kingdom*

[2] *School of Chemistry, University of Bristol, Cantock's Close, Bristol, BS8 1TS, United Kingdom*

[*] Authors to whom correspondence should be addressed.

[‡] *Now at: Department of Atmospheric and Oceanic Sciences, McGill University, 805 Sherbrooke Street West, Montreal, Quebec, H3A 0B9, Canada*

Contents:

SI1: Description of Cavity Ring-Down Spectrometer

SI2: Description of Background Subtraction Method

SI3: Generation of Spectra with Wavelength-Invariant Refractive Index

SI4: Fitting of Cavity Ring-Down Spectroscopy Data

SI5: Precision of Retrieved Radius and Effective Oscillator Parameters

SI6: Retrieval Method for Effective Oscillator Parameters of Ammonium Sulfate

SI1: Description of Cavity Ring-Down Spectrometer

Light from a 532 nm laser (Opus 532, Laser Quantum) is passed through an acousto-optic modulator (AOM, ASM-1101LA65, Intra-Action,). The first-order diffraction peak from the AOM is passed through a mode-matching lens into a linear optical cavity formed by two highly reflective mirrors (Layertec) with reflectivities greater than 0.9999 at the 532 nm wavelength and radii of curvature of 1 m, separated by a distance $L = 0.6$ m. The second cavity mirror is mounted on a piezo ring actuator (Piezomechanik), driven with a triangular waveform from a signal generator that oscillates the position of the mirror at 60 Hz. Build-up of light inside the cavity is monitored using a photodiode (D101, RedWave Labs), and the intensity escaping the cavity measured as an output voltage that is sent to both a Compuscope 14-bit digitizer (Octopus CS8322, GaGe) and a digital delay generator (Sapphire 9200, Quantum Composers). The digital delay generator triggers when the leading edge of the photodiode voltage reaches a threshold value (typically ~1.3 V). Subsequently, the digital delay generator sends a 5 V TTL pulse to the AOM, causing the first-order diffraction beam to be switched off, initiating ring-down decay. Ring-down data are measured at between 25 and 40 Hz and averaged to an acquisition rate of 1 Hz.

SI2: Description of Background Subtraction Method

We use in-house-written Python code to perform adaptive baseline subtraction on experimental spectra. Our approach is broadly similar to those used by Kohli *et al.* and Barker *et al.*[1, 2] The specifics of our process are demonstrated for a single measured spectrum in Figure S1. The procedure begins by generating a reasonable guess of the background intensity profile. We do this by taking the average intensity value of each pixel across the experiment. The logic underlying this step is that, while in any given frame some pixels will see significantly enhanced scattering from Mie resonances, as the particle changes size (and potentially refractive index) over the course of the experiment these resonances shift, averaging out the resonant enhancements across the

whole wavelength range and revealing the underlying background shape (red line in Figure S1(a)). Once the shape of the background is determined, it can then be scaled on a frame-by-frame basis. We found that a single scaling factor applied to the whole spectrum was generally insufficient. Therefore, we apply wavelength-dependent scaling factors. The scaling factors are determined by breaking the spectrum into a series of wavelength bins chosen by the user. It is important to choose the width of these bins such that the bin width is greater than the width of any resonant features in the spectrum. As such, we used different bin widths as appropriate for a given experiment. The experimental spectrum (black line in Figure S1(a)) is divided by the background shape (red line). We reason that within each wavelength bin, the minimum value of this ratio is the factor by which the background needs to be scaled. Thus, we find the minimum value of the ratio between the experimental spectrum and the background shape (black and red lines in Figure S1(a), respectively) in each bin (black circles in Figure S1(b)) and interpolate linearly between these (red line in Figure S1(b)) to ensure smooth variation across the spectrum. We then multiply the background shape by the wavelength-dependent ratio shown in Figure S1(b) to get the background for the frame (blue line in Figure S1(a)). The final spectrum is determined by dividing the experimental spectrum by the background spectrum. The reason for this is that the scattering intensity at a given wavelength scales linearly with the incident intensity; therefore, dividing by the background intensity correctly accounts for variations in incident irradiance as a function of wavelength.

A subtle but consequential assumption made here is that the scattered intensity at the minimum in each wavelength bin is equal. In general this is untrue, as BLS spectra have a slowly varying baseline as a function of wavelength, as well as the series of Mie resonances. Thus, performing the background subtraction as described removes the slowly varying baseline present in measured

BLS spectra, leaving only their Mie resonances. Therefore, the slowly varying baseline must also be subtracted from synthetic spectra to ensure accurate comparison with experimental spectra during fitting. The baseline subtraction is achieved by a similar method to that used for measured spectra. We take the same wavelength bins used for the experimental spectra, find the minimum value of the theoretical spectrum in each bin, and interpolate linearly between these points to determine the baseline value as a function of wavelength. The interpolated baseline values are then subtracted to yield only the Mie resonances of the synthetic spectrum, as required for comparison with the Mie resonances of experimental spectra.

SI3: Generation of Spectra with Wavelength-Invariant Refractive Index

The approximate parameter retrieval method described in Section 3.1 of the main text requires the generation of BLS spectra for all combinations of a grid of radius values, denoted r_i, and a grid of refractive index values, denoted n_i. Our recent publication describes an algorithm that calculates BLS spectra quickly and efficiently.[3] However, in the case we are concerned with here, there are further opportunities for computational efficiency that do not apply in the more general cases for which the algorithm in our previous work was intended. As outlined in several places, the calculation of scattering spectra requires evaluation of the integral:[3-5]

$$I(x,n) = \frac{1}{k^2}\int_0^{2\pi}\int_0^{\theta_\mathrm{max}} S_{11}(x,n,\theta_\mathrm{s},\varphi_\mathrm{s})\,\sin\theta_\mathrm{s}\,d\theta_\mathrm{s}\,d\varphi_\mathrm{s} \qquad \text{(SI1)}$$

where $I(x,n)$ is the scattered intensity for a particle of refractive index n and size parameter $x = 2\pi r/\lambda$, where r is the particle radius and λ is the wavelength of incident light. $k = 2\pi/\lambda$ is the angular wavenumber of the incident light, $\theta_\mathrm{max}, \theta_\mathrm{s}$ and φ_s are scattering angles defined by the experimental geometry, and S_{11} is an element of the Müller matrix which relates incident and scattered intensities.

To calculate spectra with constant refractive index efficiently, we note two things: (i) there is considerable overlap in the size parameters for which equation (SI1) needs to be evaluated between adjacent values of r_i, and (ii) n_i being constant means that, for x values within the overlapping size parameter regime in (i), the value of the double integral in equation (SI1) does not change for different r_i values. Points (i) and (ii) are easily illustrated with an example. For the wavelength range used in our experiments, i.e. 380–800 nm, taking r_i = 1.000 μm would require evaluation of equation (SI1) for x values spanning 7.85–16.5, and for r_i = 1.010 would require x spanning 7.93–16.7. When n is constant, the double integral values for x spanning 7.93–16.5 are identical. Thus, we can avoid a substantial number of repeated calculations by evaluating the double integral for x values spanning 7.85–16.7 and then interpolate to the relevant values for a given value of r_i. To extend this to all necessary values of x, we denote the minimum and maximum values of r_i as r_{min} and r_{max}, respectively. The necessary size parameter range for which the double integral in equation (SI1) needs to be evaluated spans from $x_{min} = 2\pi r_{min}/\lambda_{max}$ and $x_{max} = 2\pi r_{max}/\lambda_{min}$, where λ_{min} and λ_{max} are the shortest and longest wavelengths in the experimental spectra. Spectra for each combination of r_i and n_i are then evaluated by the following procedure:

1. Evaluate double integral of equation (SI1) for a given n_i for values spanning x_{min} to x_{max}
2. Interpolate values from step 1 to those needed for a given r_i, i.e. at values $x = 2\pi r_i/\lambda$
3. Multiply values from step 2 by $1/k^2$

For typical particle sizes used in our experiments, we find that the resolution required to accurately reproduce Mie resonances in x for step 1 is 0.025. Experiments using larger particles will likely require greater resolution to adequately capture the fine resonance structure seen in their BLS spectra.

SI4: Fitting of Cavity Ring-Down Spectroscopy Data

Measured ring-down times are converted to extinction cross-sections (σ_{ext}) using:[6]

$$\sigma_{\mathrm{ext}}(r,n) = \frac{\pi L w_0^2}{2c}\left(\frac{1}{\tau} - \frac{1}{\tau_0}\right) \quad \text{(SI2)}$$

with L the cavity length (0.6 m), w_0 the beam waist of the intracavity CRDS beam, c the speed of light, τ the measured ring-down time, and τ_0 the empty cavity ring-down time that is measured for ~5 minutes after the particle leaves the optical trap and is typically 35–40 μs in our experiments. A two-dimensional grid search is used to retrieve w_0 and n by maximizing the coefficient of determination:

$$R^2 = 1 - \frac{\sum_{\mathrm{i}}(\sigma_{\mathrm{i}}^{\mathrm{m}}(w_0) - \sigma_{\mathrm{i}}^{\mathrm{t}}(n))^2}{\sum_{\mathrm{i}}\left(\sigma_{\mathrm{i}}^{\mathrm{m}}(w_0) - \overline{\sigma^{\mathrm{m}}(w_0)}\right)^2} \quad \text{(SI3)}$$

in which i is the index of a given data point, σ^{m} (calculated using a chosen value of w_0) is the measured value of σ_{ext}, σ^{t} (calculated using a chosen value of n) is the theoretical value of σ_{ext}, and the bar indicates the mean value of the measured cross-sections. For the calculation of σ^{t}, a particle radius is needed. The radius values are taken from the BLS retrievals. The grid search proceeds in three stages with increasing resolution, first searching w_0 and n with resolutions of 2 μm and 0.01, respectively. The next stage searches around the maximum of the first stage with resolutions of 0.5 μm and 0.001, and the final stage searches around the second-stage maximum with resolutions of 0.1 μm and 0.0001 in w_0 and n, respectively.

SI5: Precision of Retrieved Radius and Effective Oscillator Parameters

Figure S4 investigates the precision of the retrieved parameters, and by extension the precision of the retrieved refractive index. Figure S4(a) shows the fractional standard deviation in the radius for each of the experiments shown in Figure 6 of the main text. The standard deviation is calculated relative to a smoothed fit of the retrieved radii using a Savitzky-Golay filter then divided by the

average radius in the experiment to find the fractional standard deviation. Fractional standard deviations in A and ν_0 are shown in Figure S4(b). In both cases, fractional uncertainties are on the order of 2 %, with uncertainties in A tending to be higher. The average fractional uncertainty in A is 2.6 %, compared to 1.6 % for ν_0. An example of how uncertainties in oscillator parameters propagate into n is shown in Figure S4(c). Uncertainty calculations in this case are performed by calculating n using $\bar{A} + \sigma_A$ and $\bar{\nu_0} + \sigma_\nu$, where σ_A and σ_ν are the standard deviations in each oscillator parameter and then subtracting n calculated using $\bar{A}$ and $\bar{\nu_0}$. As can be seen from this example, the wavelength dependence of the uncertainty is weak, and is around 0.004 across the full wavelength range measured. Taking the uncertainties in Figure 6(b) of the main text as representative values, the mean precision of our refractive index retrievals is 0.004, corresponding to a fractional uncertainty of 0.27 % on the mean value of n obtained from BLS retrievals.

SI6: Retrieval Method for Effective Oscillator Parameters of Ammonium Sulfate

To disentangle the contributions of water and ammonium sulfate to the measured refractive index, we recognize that ammonium sulfate is nonvolatile and therefore the mass of ammonium sulfate in the particle remains constant as the particle radius changes. This mass, m_{AS}, is simply the mass fraction of ammonium sulfate, w_{AS}, in the droplet multiplied by the total mass of the particle. The total mass of the particle is given by the product of its density, ρ, and volume, $V = 4\pi r^3/3$, giving:

$$m_{\mathrm{AS}} = \frac{4\pi}{3} w_{\mathrm{AS}} \rho r^3 \tag{SI4}$$

Assuming an initial value for w_{AS}, denoted w_{i}, allows calculation of m_{AS}, which is then used to predict how particle composition changes as a function of radius. This information can then be used in conjunction with equation (2) of the main text to predict the wavelength-dependent refractive index of the mixed particle, so long as effective oscillator parameters for each

component are known. In practice, we use the parameterization of Daimon and Masumura to calculate the refractive index of water,[7] meaning equation (2) of the main text is modified to:

$$n = 1 + \frac{2}{\pi}\frac{\phi_{\mathrm{s}} A_{\mathrm{s}} \nu_{\mathrm{s}}}{(\nu_{\mathrm{s}}^2 - \nu^2)} + \phi_{\mathrm{w}}(n_{\mathrm{w}}(\nu) - 1) \quad \text{(SI5)}$$

with $n_{\mathrm{w}}(\nu)$ calculated using the parameterization from reference [7]. Optimal values of w_{i}, A_{s} and ν_{s} are found using a differential evolution algorithm[8] which minimizes the sum-of-squares error between predicted and BLS-retrieved values of refractive index across the whole dataset. For a given combination of w_{i}, A_{s} and ν_{s}, w_{i} is used in conjunction with equation (SI4) to predict the composition of the particle and the relative densities of ammonium sulfate and water at each measured radius, calculated using the parameterization of Tang *et al.* as described in the main text.[9] Then, candidate values of A_{s} and ν_{s} are used along with the relative densities and equation (SI5) to predict the wavelength-dependent refractive index for each spectrum, $n^{\mathrm{pred}}(\lambda)$. The measured refractive index, $n^{\mathrm{meas}}(\lambda)$, is calculated for each frame using the fitted values of A_{e} and ν_{e} from the BLS retrievals reported above. The sum-of-squares error between the predicted measured values is calculated by:

$$SSE = \sum_{\mathrm{j}} \sum_{\mathrm{i}} \left(n_{\mathrm{j}}^{\mathrm{meas}}(\lambda_{\mathrm{i}}) - n_{\mathrm{j}}^{\mathrm{pred}}(\lambda_{\mathrm{i}})\right)^2 \quad \text{(SI6)}$$

in which the index i sums across the wavelengths in a spectrum, and j across all the measured spectra in the experiment.

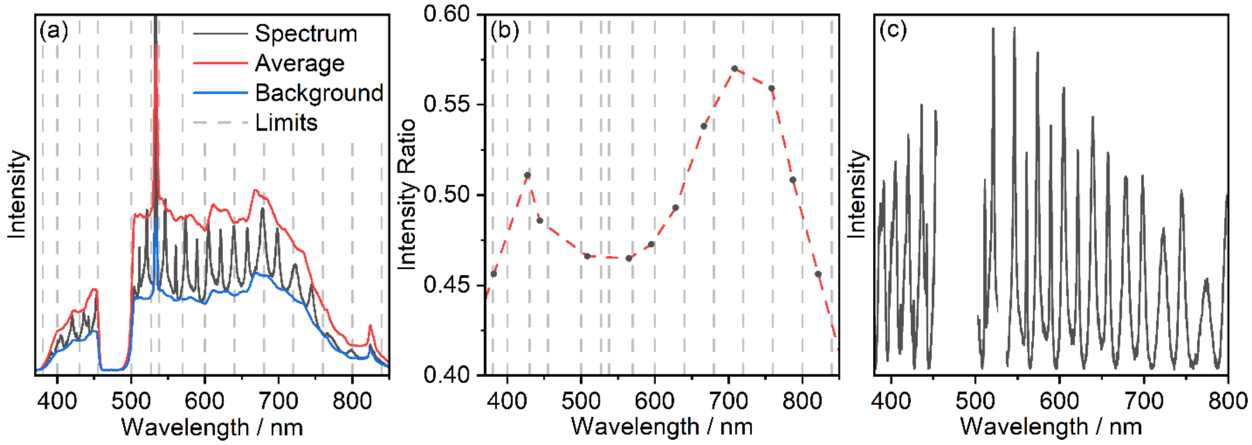

Figure S1: (a) Example of background subtraction for a single frame. The average spectrum across the full experiment is shown in red, the experimental spectrum in black and the background determined for this frame in blue. Limits of the wavelength bins used in this process are indicated by grey dashed lines. (b) Minimum ratio in each bin (black circles) between the experimental and average spectra shown in panel (a) and interpolated values (black line) used to multiply the average spectrum to get the blue spectrum in panel (a).

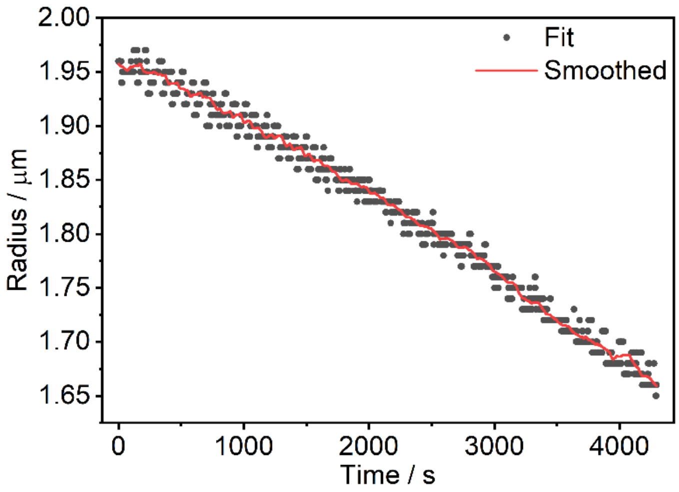

Figure S2: Example of approximate radius values (black circles) and their smoothing using a Savitzky-Golay filter (red line).

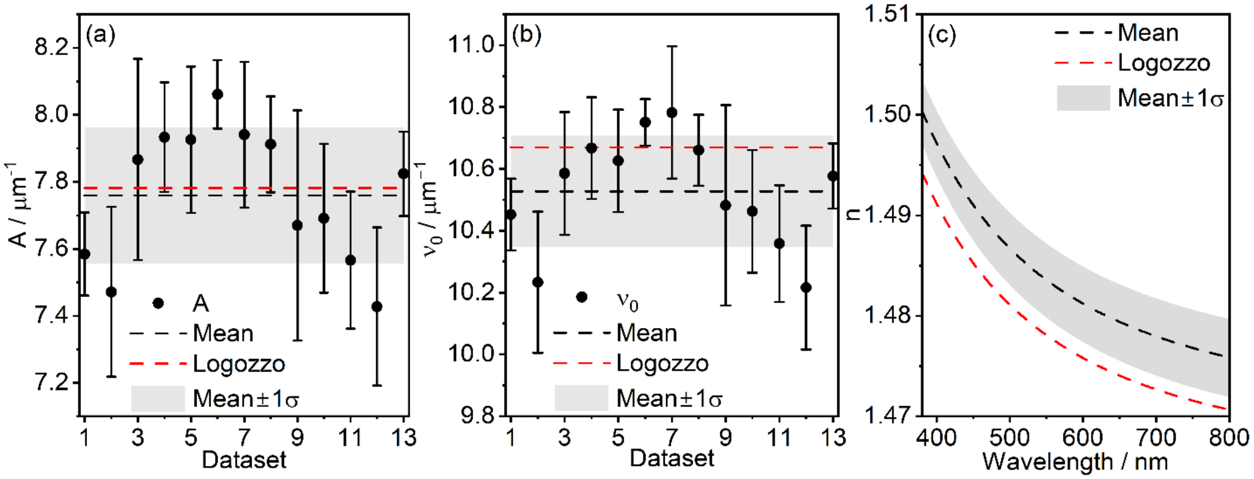

Figure S3: Oscillator parameters (a) A and (b) ν_0 (black circles) retrieved for 1,2,6-hexanetriol along with their mean (black dashed line) and standard deviation (grey shaded area (c) Wavelength-dependent refractive index (black line) and its standard deviation (grey shaded area) calculated using the mean parameters in (a) and (b). Red dashed lines are values from a previous measurement.[10]

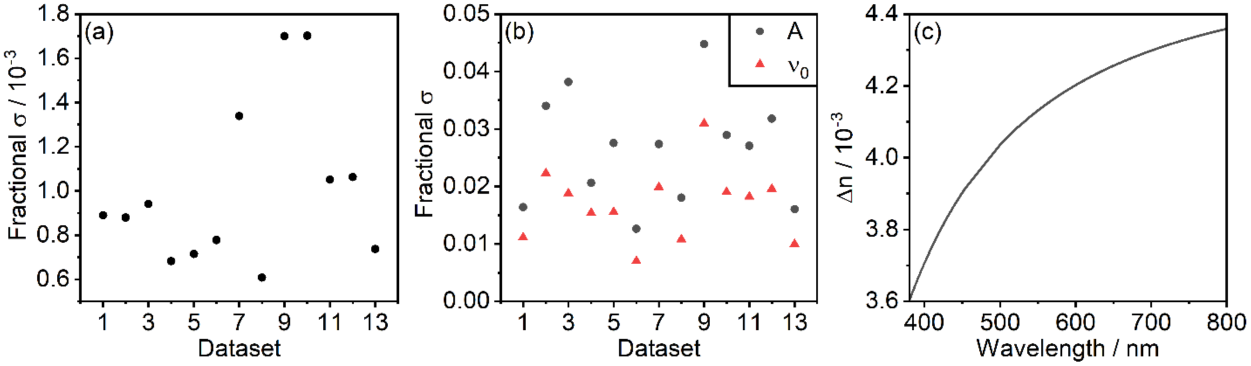

Figure S4: Fractional standard deviation in retrieved (a) radius and (b) effective oscillator parameters A (black circles) and ν_0 (red triangles) for each measurement on 1,2,6-hexanetriol. (c) An example of the wavelength-dependent refractive index uncertainty resulting from the uncertainties in A and ν_0.

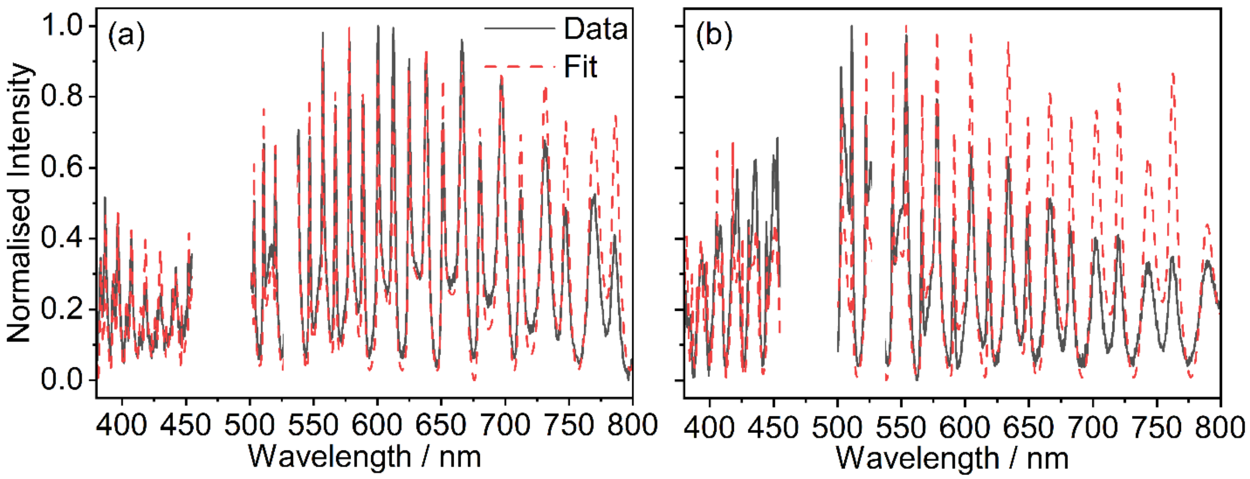

Figure S5: Example fits with (a) high ($C = 0.92$) and (b) low ($C = 0.728$) correlation values from frames 1100 and 1500 of the data in main text Figure 7, respectively.

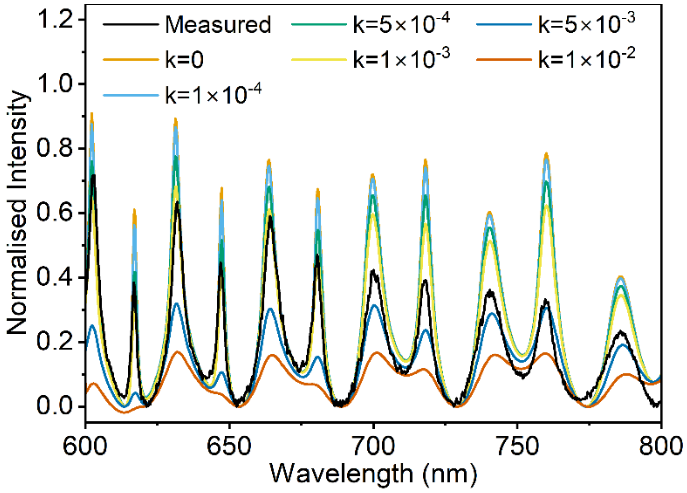

Figure S6: Normalized measured spectrum from Figure S5(b) (black) along with synthetic spectra with various values of k spanning 0–0.01 as indicated by the legend. Spectra are generated using the retrieved parameters for the measured data: r = 1.557 μm, A = 7.65 μm^{-1}, and υ_0 = 10.8 μm^{-1}. k for the synthetic spectra are constant across the wavelength range shown.

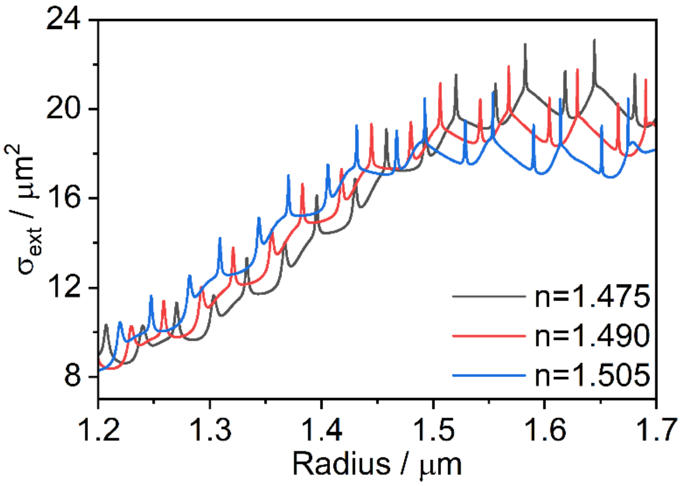

Figure S7: Example extinction cross-sections for refractive indices of 1.475 (black), 1.490 (red) and 1.505 (blue).

AUTHOR INFORMATION

Corresponding Author

* Authors to whom correspondence should be addressed.

Aidan Rafferty – *Department of Chemistry, University of Oxford, Physical and Theoretical Chemistry Laboratory, South Parks Road, Oxford, OX1 3QZ, United Kingdom*

Now at: Department of Atmospheric and Oceanic Sciences, 805 Sherbrooke Street West Montreal, Quebec, H3A 0B9, Canada

Email: aidan.rafferty@mail.mcgill.ca

Michael I. Cotterell – *Department of Chemistry, University of Oxford, Physical and Theoretical Chemistry Laboratory, South Parks Road, Oxford, OX1 3QZ, United Kingdom*

Email: michael.cotterell@chem.ox.ac.uk

Author Information

Andrew J. Orr-Ewing – *School of Chemistry, University of Bristol, Cantock's Close, Bristol, BS8 1TS, United Kingdom*

Jonathan P. Reid – *School of Chemistry, University of Bristol, Cantock's Close, Bristol, BS8 1TS, United Kingdom*

REFERENCES

(1) Kaur Kohli, R.; Davis, R. D.; Davies, J. F. Tutorial: Electrodynamic balance methods for single particle levitation and the physicochemical analysis of aerosol. *J. Aerosol. Sci.* **2023**, *174*. DOI: 10.1016/j.jaerosci.2023.106255.

(2) Barker, C. R.; King, M. D.; Ward, A. D. Separation-dependent near-field effects in Mie scattering spectra of two optically trapped aerosol droplets. *Opt. Express* **2024**, *32* (12). DOI: 10.1364/oe.520251.

(3) Rafferty, A.; Orr-Ewing, A. J.; Reid, J. P.; Cotterell, M. I. Efficient calculation of broadband light scattering spectra from spherical, homogeneous particles. *J. Quant. Spectrosc. Radiat. Transfer* **2026**, *348*. DOI: 10.1016/j.jqsrt.2025.109708.

(4) Bain, A.; Preston, T. C. Mie scattering from strongly absorbing airborne particles in a photophoretic trap. *J. Appl. Phys.* **2019**, *125* (9), 093101. DOI: 10.1063/1.5082157.

(5) Rothe, T.; Schmitz, M.; Kienle, A. Angular and spectrally resolved investigation of single particles by darkfield scattering microscopy. *J. Biomed. Opt.* **2012**, *17* (11), 117006. DOI: 10.1117/1.jbo.17.11.117006.

(6) Walker, J. S.; Carruthers, A. E.; Orr-Ewing, A. J.; Reid, J. P. Measurements of Light Extinction by Single Aerosol Particles. *J. Phys. Chem. Lett.* **2013**, *4* (10), 1748-1752. DOI: 10.1021/jz4008068.

(7) Daimon, M.; Masumura, A. Measurement of the refractive index of distilled water from the near-infrared region to the ultraviolet region. *Appl. Opt.* **2007**, *46* (18), 3811--3820. DOI: 10.1364/AO.46.003811.

(8) Rodriguez-Mier, P. *A tutorial on Differential Evolution with Python*. 2017. https://pablormier.github.io/2017/09/05/a-tutorial-on-differential-evolution-with-python/ (accessed 2025 13/10/2025).

(9) Tang, I. N.; Munkelwitz, H. R. Water activities, densities, and refractive indices of aqueous sulfates and sodium nitrate droplets of atmospheric importance. *J. Geophys. Res.: Atmos.* **1994**, *99* (D9), 18801-18808. DOI: 10.1029/94JD01345.

(10) Logozzo, A.; Preston, T. C. Temperature-Controlled Dual-Beam Optical Trap for Single Particle Studies of Organic Aerosol. *J. Phys. Chem. A* **2022**, *126* (1), 109-118. DOI: 10.1021/acs.jpca.1c09363.
